# New verifiable stationarity concepts for a class of mathematical programs with disjunctive constraints

**DOI:** 10.1080/02331934.2017.1387547

**Published:** 2017-10-12

**Authors:** Matúš Benko, Helmut Gfrerer

**Affiliations:** ^a^ Institute of Computational Mathematics, Johannes Kepler University Linz, Linz, Austria.

**Keywords:** Mathematical programs with disjunctive constraints, B-stationarity, M-stationarity, $Q_{M}$-stationarity

## Abstract

In this paper, we consider a sufficiently broad class of non-linear mathematical programs with disjunctive constraints, which, e.g. include mathematical programs with complemetarity/vanishing constraints. We present an extension of the concept of Q-stationarity which can be easily combined with the well-known notion of M-stationarity to obtain the stronger property of so-called $Q_{M}$-stationarity. We show how the property of $Q_{M}$-stationarity (and thus also of M-stationarity) can be efficiently verified for the considered problem class by computing Q-stationary solutions of a certain quadratic program. We consider further the situation that the point which is to be tested for $Q_{M}$-stationarity, is not known exactly, but is approximated by some convergent sequence, as it is usually the case when applying some numerical method.

## 1. Introduction

In this paper, we consider the following *mathematical program with disjunctive constraints* (MPDC)

(1) $$\begin{array}{cc} \min_{x\in R^{n}} & f(x)\\ \;\text{subject to} & F_{i}\left(x\right)\in D_{i}:=\overset{K_{i}}{\underset{j=1}{⋃}}D_{i}^{j},\;i=1,\ldots ,m_{D}, \end{array}$$

where the mappings $f:R^{n}\to R$ and $F_{i}:R^{n}\to R^{l_{i}}$, $i=1,\ldots ,m_{D}$ are assumed to be continuously differentiable and $D_{i}^{j}\subset R^{l_{i}}$, $j=1,\ldots ,K_{i}$, $i=1,\ldots ,m_{D}$ are convex polyhedral sets.

Denoting $m:=\sum_{i=1}^{m_{D}}l_{i}$,

(2) $$\begin{array}{c} F:=\left(F_{1},\ldots ,F_{m_{D}}\right):R^{n}\to R^{m},\;D:=\prod_{i=1}^{m_{D}}D_{i} \end{array}$$

we can rewrite the MPDC (1) in the form

(3) $$\begin{array}{c} \min_{x\in R^{n}}f\left(x\right)\;\;\text{subject to}\;F\left(x\right)\in D. \end{array}$$

It is easy to see that *D* can also be written as the union of $\prod_{i=1}^{m_{D}}K_{i}$ convex polyhedral sets by

(4) $$\begin{array}{c} D=\underset{\nu \in J}{⋃}D\left(\nu \right)\;\;\text{with}\;J:=\prod_{i=1}^{m_{D}}\left\{1,\ldots ,K_{i}\right\},\;D\left(\nu \right):=\prod_{i=1}^{m_{D}}D_{i}^{\nu_{i}}. \end{array}$$

As an example for MPDC consider a *mathematical program with complementarity constraints* (MPCC) given by

(5) $$\begin{array}{cc} \min_{x\in R^{n}} & f(x)\\ \;\text{subject to} & g_{i}\left(x\right)\leq 0,\;i=1,\ldots m_{I},\\ & h_{i}\left(x\right)=0,\;i=1,\ldots m_{E},\\ & G_{i}\left(x\right)\geq 0,\;H_{i}\left(x\right)\geq 0,G_{i}\left(x\right)H_{i}\left(x\right)=0,\;i=1,\ldots m_{C} \end{array}$$

with $f:R^{n}\to R$, $g_{i}:R^{n}\to R$, $i=1,\ldots ,m_{I}$, $h_{i}:R^{n}\to R$, $i=1,\ldots ,m_{E}$, $G_{i},H_{i}:R^{n}\to R$, $i=1,\ldots ,m_{C}$. This problem fits into our setting (1) with $m_{D}=m_{C}+1$,

$$\begin{array}{cc} F_{1} & ={\left(g_{1},\ldots ,g_{m_{I}},h_{1}\ldots ,h_{m_{E}}\right)}^{T},\;D_{1}^{1}=R_{-}^{m_{I}}\times {\left\{0\right\}}^{m_{E}},\;l_{1}=m_{I}+m_{E},\;K_{1}=1\\ F_{i+1} & ={\left(-G_{i},-H_{i}\right)}^{T},\;D_{i+1}^{1}=\left\{0\right\}\times R_{-},\;D_{i+1}^{2}=R_{-}\times \left\{0\right\},\;l_{i+1}=K_{i+1}=2,\;i=1,\ldots ,m_{C}. \end{array}$$

MPCC is known to be a difficult optimization problem, because, due to the complementarity constraints $G_{i}\left(x\right)\geq 0$, $H_{i}\left(x\right)\geq 0$, $G_{i}\left(x\right)H_{i}\left(x\right)=0$, many of the standard constraint qualifications of nonlinear programming are violated at any feasible point. Hence, it is likely that the usual Karush–Kuhn–Tucker conditions fail to hold at a local minimizer and various first-order optimality conditions such as Abadie (A-), Bouligand (B-), Clarke (C-), Mordukhovich (M-) and Strong (S-) stationarity conditions have been studied in the literature [1–9].

Another prominent example is the *mathematical program with vanishing constraints* (MPVC)

(6) $$\begin{array}{cc} \min_{x\in R^{n}} & f(x)\\ \;\text{subject to} & g_{i}\left(x\right)\leq 0,\;i=1,\ldots m_{I},\\ & h_{i}\left(x\right)=0,\;i=1,\ldots m_{E},\\ & H_{i}\left(x\right)\geq 0,\;G_{i}\left(x\right)H_{i}\left(x\right)\leq 0,\;i=1,\ldots m_{V} \end{array}$$

with $f:R^{n}\to R$, $g_{i}:R^{n}\to R$, $i=1,\ldots ,m_{I}$, $h_{i}:R^{n}\to R$, $i=1,\ldots ,m_{E}$, $G_{i},H_{i}:R^{n}\to R$, $i=1,\ldots ,m_{V}$. Again, the problem MPVC can be written in the form (1) with $m_{D}=m_{V}+1$, $F_{1}$, $D_{1}^{1}$ as in the case of MPCC and

$$\begin{array}{c} F_{i+1}={\left(-H_{i},G_{i}\right)}^{T},\;D_{i+1}^{1}=\left\{0\right\}\times R,\;D_{i+1}^{2}=R_{-}^{2},\;l_{i+1}=K_{i+1}=2,\;i=1,\ldots ,m_{V}. \end{array}$$

Similar as in the case of MPCC, many of the standard constraint qualifications of non-linear programming can be violated at a local solution of (6) and a lot of stationarity concepts have been introduced. For a comprehensive overview for MPVC we refer to [10] and the references therein.

However, when we do not formulate MPCC or MPVC as a non-linear program but as a disjunctive program MPDC, then first-order optimality conditions can be formulated which are valid under weak constraint qualifications. We know that a local minimizer is always B-stationary, which geometrically means that no feasible descent direction exists, or, in a dual formulation, that the negative gradient of the objective belongs to the regular normal cone of the feasible region, cf. [11, Theorem 6.12]. The difficult task is now to estimate this regular normal cone. For this regular normal cone always a lower inclusion is available, which yields so-called S-stationarity conditions. For an upper estimate, one can use the limiting normal cone which results in the so-called M-stationarity conditions. The notions of S-stationarity and M-stationarity have been introduced in [12] for general programs (3). S-stationarity always implies B-stationarity, but it requires some strong qualification condition on the constraints which is too restrictive. On the other hand, M-stationarity requires only some weak constraint qualification but it does not preclude the existence of feasible descent directions. Further, it is not known in general how to efficiently verify the M-stationarity conditions, since the description of the limiting normal cone involves some combinatorial structure which is not known to be resolved without enumeration techniques. These difficulties in verifying M-stationarity have also some impact for numerical solution procedures. E.g. for many algorithms for MPCC it cannot be guaranteed that a limit point is M-stationary, cf. [13].

In the recent paper [14], we derived another upper estimate for the regular normal cone yielding so-called Q-stationarity conditions. Q-stationarity has the advantage over S-stationarity that it does not require such unnecessarily strong constraint qualification conditions. Q-stationarity can be easily combined with M-stationarity to obtain so-called $Q_{M}$-stationarity which is stronger than M-stationarity. This is one of the advantages of $Q_{M}$-stationarity: there are several stationarity notions, in particular in the MPCC literature, like M-, C-, A- and weak stationarity, which are valid under weak constraint qualification conditions. M-stationarity is known to be the strongest stationarity concept and we even improve M-stationarity by $Q_{M}$-stationarity.

For the disjunctive formulations of the problems MPCC and MPVC the Q- and $Q_{M}$-stationarity conditions have been worked out in detail in [14]. In this paper, we extend this approach to the general problem MPDC. We show that under a qualification condition which ensures S-stationarity of local minimizers, Q-stationarity and S-stationarity are equivalent. Further, we prove that under some weak constraint qualification every local minimizer of MPDC is a $Q_{M}$-stationary solution and we provide an efficient algorithm for verifying $Q_{M}$-stationarity of some feasible point. More exactly, this algorithm either proves the existence of some feasible descent direction, i.e. the point is not B-stationary, or it computes multipliers fulfilling the $Q_{M}$-stationarity condition. To this end, we consider quadratic programs with disjunctive constraints (QPDC), i.e. the objective function *f* in MPDC is a convex quadratic function and the mappings $F_{i}$, $i=1,\ldots ,m_{D}$ are linear. We propose a basic algorithm for QPDC, which either returns a Q-stationary point or proves that the problem is unbounded. Further, we show that M-stationarity for MPDC is related with Q-stationarity of some QPDC and the combination of the two parts yields the algorithm for verifying $Q_{M}$-stationarity. This algorithm does not rely on enumeration techniques and this is another big advantage of the concepts of Q- and $Q_{M}$-stationarity.

Our approach is well suited to the MPDC (1) when all the numbers $K_{i}$, $i=1,\ldots ,m_{D}$ are small or of moderate size. Our disjunctive structure is not induced by integral variables like, e.g. in [15]. It is also not related to the approach of considering the convex hull of a family of convex sets like in [16,17].

The outline of the paper is as follows. In Section 2, we recall some basic definitions from variational analysis and discuss various stationarity concepts. In Section 3, we introduce the concepts of Q- and $Q_{M}$-stationarity for general optimization problems. These concepts are worked out in more detail for MPDC in Section 4. In Section 5, we consider quadratic programs with disjunctive linear constraints. We present a basic algorithm for solving such problems, which either return a Q-stationary solution or prove that the problem is not bounded below. In the next section, we demonstrate how this basic algorithm can be applied to a certain quadratic program with disjunctive linear constraints in order to verify M-stationarity or $Q_{M}$-staionarity of a point or to compute a descent direction. In the last Section 7, we present some results for numerical methods for solving MPDC which prevent convergence to non M-stationary and non-$Q_{M}$-stationary points.

Our notation is fairly standard. In Euclidean space $R^{n}$ we denote by ‖·‖ and ⟨·,·⟩ the Euclidean norm and scalar product, respectively, whereas we denote by ${\left\|u\right\|}_{\infty }:=\max\{|u_{i}\left|\;|\;i=1,\ldots ,n\right\}$ the maximum norm. The closed ball around some point *x* with radius *r* is denoted by B(x,r). Given some cone $Q\subset R^{n}$, we denote by $Q^{\circ }:=\left\{q^{*}\in R^{n}\;|\;\left\langle q^{*},q\right\rangle \leq 0\forall q\in Q\right\}$ its polar cone. By d(x,A):=inf{‖x--y‖|y∈A} we refer to the usual distance of some point *x* to a set *A*. We denote by $0^{+}C$ the recession cone of a convex set *C*.

## 2. Preliminaries

For the reader’s convenience, we start with several notions from variational analysis. Given a set $\Omega \subset R^{n}$ and a point $\overset{¯}{z}\in \Omega$, the cone

$$\begin{array}{c} T_{\Omega }\left(\overset{¯}{z}\right)=\{w\;|\;\exists w_{k}\to w,t_{k}↓0\;\text{with}\overset{¯}{z}+t_{k}w_{k}\in \Omega \} \end{array}$$

is called the (Bouligand/Severi) *tangent/contingent cone* to Ω at $\overset{¯}{z}$. The (Fréchet) *regular normal cone* to Ω at $\overset{¯}{z}\in \Omega$ can be equivalently defined either by

$$\begin{array}{c} {\hat{N}}_{\Omega }\left(\overset{¯}{z}\right):=\{v^{*}\in R^{d}\;|\;\underset{z\overset{\Omega }{\to }\overset{¯}{z}}{lim sup}\frac{\left\langle v^{*},z-\overset{¯}{z}\right\rangle }{\left\|z-\overset{¯}{z}\right\|}\leq 0\}, \end{array}$$

where $z\overset{\Omega }{\to }\overset{¯}{z}$ means that $z\to \overset{¯}{z}$ with z∈Ω, or as the dual/polar to the contingent cone, i.e. by

$$\begin{array}{c} {\hat{N}}_{\Omega }\left(\overset{¯}{z}\right):=T_{\Omega }{\left(\overset{¯}{z}\right)}^{\circ }. \end{array}$$

For convenience, we put $N_{\Omega }\left(\overset{¯}{z}\right):=\emptyset$ for $\overset{¯}{z}\notin \Omega$. Further, the (Mordukhovich) *limiting/basic normal cone* to Ω at $\overset{¯}{z}\in \Omega$ is given by

$$N_{\Omega }\left(\overset{¯}{z}\right):=\{w^{*}\in R^{d}\;|\;\exists \;z_{k}\to \overset{¯}{z},\;w_{k}^{*}\to w^{*}\;\;\text{with}\;w_{k}^{*}\in {\hat{N}}_{\Omega }\left(z_{k}\right)\;\;\text{for all}\;k\}.$$

If Ω is convex, then both the regular and the limiting normal cones coincide with the normal cone in the sense of convex analysis. Therefore, we will use in this case the notation $N_{\Omega }$.

Consider now the general mathematical program

(7) $$\begin{array}{c} \min_{x\in R^{n}}f\left(x\right)\;\;\text{subject to}\;F\left(x\right)\in D \end{array}$$

where $f:R^{n}\to R$, $F:R^{n}\to R^{m}$ are continuously differentiable and $D\subset R^{m}$ is a closed set. Let

(8) $$\begin{array}{c} \Omega :=\{x\in R^{n}\;|\;F\left(x\right)\in D\} \end{array}$$

denote the feasible region of the program (7). Then a necessary condition for a point $\overset{¯}{x}\in \Omega$ being locally optimal is

(9) $$\begin{array}{c} \left\langle \nabla f\left(\overset{¯}{x}\right),u\right\rangle \geq 0\;\forall u\in T_{\Omega }\left(\overset{¯}{x}\right), \end{array}$$

which is the same as

(10) $$\begin{array}{c} -\nabla f\left(\overset{¯}{x}\right)\in {\hat{N}}_{\Omega }\left(\overset{¯}{x}\right), \end{array}$$

cf. [11, Theorem 6.12]. The main task of applying this first-order optimality condition now is the computation of the regular normal cone ${\hat{N}}_{\Omega }\left(\overset{¯}{x}\right)$ which is very difficult for nonconvex *D*.

We always have the inclusion

(11) $$\begin{array}{c} \nabla F{\left(\overset{¯}{x}\right)}^{T}{\hat{N}}_{D}\left(F\left(\overset{¯}{x}\right)\right)\subset {\hat{N}}_{\Omega }\left(\overset{¯}{x}\right), \end{array}$$

but equality will hold in (11) for nonconvex sets *D* only under comparatively strong conditions, e.g. when $\nabla F(\overset{¯}{x})$ is surjective, see [11, Exercise 6.7]. The following weaker sufficient condition for equality in (11) uses the notion of metric subregularity.

Definition 1: A multifunction $\Psi :R^{n}⇉R^{m}$ is called *metrically subregular* at a point $\left(\overset{¯}{x},\overset{¯}{y}\right)$ of its graph gphΨ with modulus κ>0, if there is a neighborhood *U* of $\overset{¯}{x}$ such that

$$\begin{array}{c} d\left(x,\Psi^{-1}\left(\overset{¯}{y}\right)\right)\leq \kappa d\left(\overset{¯}{y},\Psi \left(x\right)\right)\;\forall x\in U. \end{array}$$

Theorem 1: [18, Theorem 4]]Let Ω be given by (8) and $\overset{¯}{x}\in \Omega$. If the multifunction x⇉F(x)-D is metrically subregular at $\left(\overset{¯}{x},0\right)$ and if there exists a subspace $L\subset R^{m}$ such that

(12) $$\begin{array}{c} T_{D}\left(F\left(\overset{¯}{x}\right)\right)+L\subset T_{D}\left(F\left(\overset{¯}{x}\right)\right) \end{array}$$

and

(13) $$\begin{array}{c} \nabla F\left(\overset{¯}{x}\right)R^{n}+L=R^{m}, \end{array}$$

then

$$\begin{array}{c} {\hat{N}}_{\Omega }\left(\overset{¯}{x}\right)=\nabla F{\left(\overset{¯}{x}\right)}^{T}{\hat{N}}_{D}\left(F\left(\overset{¯}{x}\right)\right). \end{array}$$

In order to state an upper estimate for the regular normal cone ${\hat{N}}_{\Omega }\left(\overset{¯}{x}\right)$ we need some constraint qualification.

Definition 2: [12, Definition 6]] Let Ω be given by (8) and let $\overset{¯}{x}\in \Omega$.

(1) We say that the *generalized Abadie constraint qualification* (GACQ) holds at $\overset{¯}{x}$ if
  (14) $$\begin{array}{c} T_{\Omega }\left(\overset{¯}{x}\right)=T_{\Omega }^{\mathrm{lin}}\left(\overset{¯}{x}\right), \end{array}$$
  where $T_{\Omega }^{\mathrm{lin}}\left(\overset{¯}{x}\right):=\left\{u\in R^{n}\;|\;\nabla F\left(\overset{¯}{x}\right)u\in T_{D}\left(F\left(\overset{¯}{x}\right)\right)\right\}$ denotes the *linearized cone*.
(2) We say that the *generalized Guignard constraint qualification* (GGCQ) holds at $\overset{¯}{x}$ if
  (15) $$\begin{array}{c} {\left(T_{\Omega }\left(\overset{¯}{x}\right)\right)}^{\circ }={\left(T_{\Omega }^{\mathrm{lin}}\left(\overset{¯}{x}\right)\right)}^{\circ }. \end{array}$$

Obviously GGCQ is weaker than GACQ, but GACQ is easier to verify by using some advanced tools of variational analysis. E.g. if the mapping x⇉F(x)-D is metrically subregular at $\left(\overset{¯}{x},0\right)$ then GACQ is fulfilled at $\overset{¯}{x}$, cf. [19, Proposition 1]. Tools for verifying metric subregularity of constraint systems can be found e.g. in [20].

Proposition 1: [14, Proposition 3]]Let Ω be given by (8), let $\overset{¯}{x}\in \Omega$ and assume that GGCQ is fulfilled, while the mapping $u⇉\nabla F\left(\overset{¯}{x}\right)u-T_{D}\left(F\left(\overset{¯}{x}\right)\right)$ is metrically subregular at (0, 0). Then

(16) $$\begin{array}{c} {\hat{N}}_{\Omega }\left(\overset{¯}{x}\right)\subset \nabla F{\left(\overset{¯}{x}\right)}^{T}N_{T_{D}\left(F\left(\overset{¯}{x}\right)\right)}\left(0\right)\subset \nabla F{\left(\overset{¯}{x}\right)}^{T}N_{D}\left(F\left(\overset{¯}{x}\right)\right). \end{array}$$

Note that we always have $N_{T_{D}\left(F\left(\overset{¯}{x}\right)\right)}\left(0\right)\subset N_{D}\left(F\left(\overset{¯}{x}\right)\right)$, see [11, Proposition 6.27]. However, if *D* is the union of finitely many convex polyhedral sets, then equality

(17) $$\begin{array}{c} N_{T_{D}\left(F\left(\overset{¯}{x}\right)\right)}\left(0\right)=N_{D}\left(F\left(\overset{¯}{x}\right)\right) \end{array}$$

holds. This is due to the fact that by the assumption on *D* there is some neighborhood *V* of 0 such that $\left(D-F\left(\overset{¯}{x}\right)\right)\cap V=T_{D}\left(F\left(\overset{¯}{x}\right)\right)\cap V$.

Let us mention that metric subregularity of the constraint mapping x⇉F(x)-D at $\left(\overset{¯}{x},0\right)$ does not only imply GACQ and consequently GGCQ, but also metric subregularity of the mapping $u⇉\nabla F\left(\overset{¯}{x}\right)u-T_{D}\left(F\left(\overset{¯}{x}\right)\right)$ at (0, 0) with the same modulus, see [21, Proposition 2.1].

The concept of metric subregularity has the drawback that, in general, it is not stable under small perturbations. It is well known that the stronger property of metric regularity is robust.

Definition 3: A multifunction $\Psi :R^{n}⇉R^{m}$ is called *metrically regular* near a point $\left(\overset{¯}{x},\overset{¯}{y}\right)$ of its graph gphΨ with modulus κ>0, if there are neighborhoods *U* of $\overset{¯}{x}$ and *V* of $\overset{¯}{y}$ such that

$$\begin{array}{c} d\left(x,\Psi^{-1}\left(y\right)\right)\leq \kappa d\left(y,\Psi \left(x\right)\right)\;\forall \left(x,y\right)\in U\times V. \end{array}$$

The infimum of the moduli κ for which the property of metric regularity holds is denoted by

$$\begin{array}{c} \mathrm{reg}\;\Psi (\overset{¯}{x},\overset{¯}{y}). \end{array}$$

In the following proposition, we gather some well-known properties of metric regularity:

Proposition 2: Let $\overset{¯}{x}\in F^{-1}\left(D\right)$ where $F:R^{n}\to R^{m}$ is continuously differentiable and *D* is the union of finitely many convex polyhedral sets and consider the multifunctions x⇉Ψ(x):=F(x)-D and $u⇉D\Psi \left(\overset{¯}{x}\right)\left(u\right):=\nabla F\left(\overset{¯}{x}\right)u-T_{D}\left(F\left(\overset{¯}{x}\right)\right)$. Then

$$\begin{array}{c} \mathrm{reg}\;\Psi \left(\overset{¯}{x},\overset{¯}{y}\right)=\mathrm{reg}\;D\Psi \left(\overset{¯}{x}\right)\left(0,0\right)=\max\{\frac{1}{{\|\nabla F\left(\overset{¯}{x}\right)}^{T}\lambda \|}\;|\;\lambda \in N_{D}\left(F\left(\overset{¯}{x}\right)\right)=N_{T_{D}\left(F\left(\overset{¯}{x}\right)\right)}\left(0\right),\;\left\|\lambda \right\|=1\}. \end{array}$$

Moreover for every $\kappa >\mathrm{reg}\;\Psi (\overset{¯}{x},\overset{¯}{y})$ there is a neighborhood *W* of $\overset{¯}{x}$ such that for all x∈W the mapping $u⇉\nabla F\left(x\right)u-T_{D}\left(F\left(\overset{¯}{x}\right)\right)$ is metrically regular near (0, 0) with modulus κ,

(18) $$\begin{array}{c} {\|\lambda \|\leq \kappa \|\nabla F\left(x\right)}^{T}\lambda \|\;\forall \lambda \in N_{D}\left(F\left(\overset{¯}{x}\right)\right)=N_{T_{D}\left(F\left(\overset{¯}{x}\right)\right)}\left(0\right) \end{array}$$

and

$$\begin{array}{c} d\left(u,\nabla F{\left(x\right)}^{-1}T_{D}\left(F\left(\overset{¯}{x}\right)\right)\right)\leq \kappa d\left(\nabla F\left(x\right)u,T_{D}\left(F\left(\overset{¯}{x}\right)\right)\right)\;\forall u\in R^{n}. \end{array}$$

**Proof **The statement follows from [11, Exercise 9.44] together with the facts that by our assumption on *D* condition (17) holds and that $T_{D}\left(F\left(\overset{¯}{x}\right)\right)$ is a cone.

We now recall some well known stationarity concepts based on the considerations above.

Definition 4: Let $\overset{¯}{x}$ be feasible for the program (7).

(i) We say that $\overset{¯}{x}$ is *B-stationary*, if (9) or, equivalently, (10) hold.
(ii) We say that $\overset{¯}{x}$ is S-stationary, if
  $$\begin{array}{c} -\nabla f\left(\overset{¯}{x}\right)\in \nabla F{\left(\overset{¯}{x}\right)}^{T}{\hat{N}}_{D}\left(F\left(\overset{¯}{x}\right)\right). \end{array}$$
(iii) We say that $\overset{¯}{x}$ is M-stationary, if
  $$\begin{array}{c} -\nabla f\left(\overset{¯}{x}\right)\in \nabla F{\left(\overset{¯}{x}\right)}^{T}N_{D}\left(F\left(\overset{¯}{x}\right)\right). \end{array}$$

Every local minimizer of (7) is B-stationary and this stationarity concept is considered to be the most preferable one. S- and M-stationarity have been introduced in [12] as a generalization of these notions for MPCC. Using the inclusion (5) it immediately follows, that S-stationarity implies B-stationarity. However the reverse implication only holds true under some additional condition on the constraints, e.g. under the assumptions of Theorem 1. Note that there always hold the inclusions

$$\begin{array}{c} \nabla F{\left(\overset{¯}{x}\right)}^{T}{\hat{N}}_{D}\left(F\left(\overset{¯}{x}\right)\right)\subset (T_{\Omega }^{\mathrm{lin}}\left(\overset{¯}{x}\right){)}^{\circ }\subset (T_{\Omega }\left(\overset{¯}{x}\right){)}^{\circ }={\hat{N}}_{\Omega }\left(\overset{¯}{x}\right). \end{array}$$

In order that a B-stationary point is also S-stationarity, both inclusions must be fulfilled with equality, i.e. besides the GGCQ $(T_{\Omega }^{\mathrm{lin}}\left(\overset{¯}{x}\right){)}^{\circ }=(T_{\Omega }\left(\overset{¯}{x}\right){)}^{\circ }$ which allows to replace the tangent cone by the linearized tangent cone, we need another constraint qualification condition ensuring $\nabla F{\left(\overset{¯}{x}\right)}^{T}{\hat{N}}_{D}\left(F\left(\overset{¯}{x}\right)\right)=(T_{\Omega }^{\mathrm{lin}}\left(\overset{¯}{x}\right){)}^{\circ }$ like the conditions (12) and (13). It is well known that this additional condition is much more restrictive than the usual constraint qualifications allowing the linearization of the problem like metric (sub)regularity of the constraint mapping F(·)-D. Thus in the general case one cannot expect that a local minimizer is also S-stationary. This is the reason why other stationarity concepts like M-stationarity have also to be considered.

A B-stationary point is M-stationary under the very weak assumptions of Proposition 1. However, the inclusion ${\hat{N}}_{\Omega }\left(\overset{¯}{x}\right)\subset \nabla F{\left(\overset{¯}{x}\right)}^{T}N_{D}\left(F\left(\overset{¯}{x}\right)\right)$ can be strict, implying that a M-stationary point $\overset{¯}{x}$ needs not to be B-stationary. Hence, M-stationarity does eventually not preclude the existence of feasible descent directions, i.e. directions $u\in T_{\Omega }\left(\overset{¯}{x}\right)$ with $\langle \nabla f(\overset{¯}{x}),u\rangle <0$.

## On Q- and $Q_{M}$-stationarity

3.

In this section, we consider an extension of the concept of Q-stationarity as introduced in the recent paper [14]. Q-stationarity is based on the following simple observation.

Consider the general program (7), assume that GGCQ holds at the point $\overset{¯}{x}\in \Omega$ and assume that we are given *K* convex cones $Q_{i}\subset T_{D}\left(F\left(\overset{¯}{x}\right)\right)$, i=1,…,K. Then for each i=1,…,K we obviously have $T_{\Omega }^{\mathrm{lin}}\left(\overset{¯}{x}\right)=\nabla F{\left(\overset{¯}{x}\right)}^{-1}T_{D}\left(F\left(\overset{¯}{x}\right)\right)⊃\nabla F{\left(\overset{¯}{x}\right)}^{-1}Q_{i}$ implying

$$\begin{array}{c} {\hat{N}}_{\Omega }\left(\overset{¯}{x}\right)={\left(T_{\Omega }^{\mathrm{lin}}\left(\overset{¯}{x}\right)\right)}^{\circ }\subset {\left(F{\left(\overset{¯}{x}\right)}^{-1}Q_{i}\right)}^{\circ }. \end{array}$$

If we further assume that ${\left(F{\left(\overset{¯}{x}\right)}^{-1}Q_{i}\right)}^{\circ }=\nabla F{\left(\overset{¯}{x}\right)}^{T}Q_{i}^{\circ }$ and by taking into account, that by [14, Lemma 1] we have

$$\begin{array}{c} \left(\nabla F{\left(\overset{¯}{x}\right)}^{T}S_{1}\right)\cap \left(\nabla F{\left(\overset{¯}{x}\right)}^{T}S_{2}\right)=\nabla F{\left(\overset{¯}{x}\right)}^{T}(S_{1}\cap \left(\ker\nabla F{\left(\overset{¯}{x}\right)}^{T}+S_{2}\right)) \end{array}$$

for arbitrary sets $S_{1},S_{2}\subset R^{m}$, we obtain

$$\begin{array}{cc} {\hat{N}}_{\Omega }\left(\overset{¯}{x}\right) & \subset \overset{K}{\underset{i=1}{⋂}}\nabla F{\left(\overset{¯}{x}\right)}^{T}Q_{i}^{\circ }=\nabla F{\left(\overset{¯}{x}\right)}^{T}(Q_{1}^{\circ }\cap \left(\ker\nabla F{\left(\overset{¯}{x}\right)}^{T}+Q_{2}^{\circ }\right))\cap \overset{K}{\underset{i=3}{⋂}}\nabla F{\left(\overset{¯}{x}\right)}^{T}Q_{i}^{\circ }\\ & =\nabla F{\left(\overset{¯}{x}\right)}^{T}(Q_{1}^{\circ }\cap \left(\ker\nabla F{\left(\overset{¯}{x}\right)}^{T}+Q_{2}^{\circ }\right)\cap \left(\ker\nabla F{\left(\overset{¯}{x}\right)}^{T}+Q_{3}^{\circ }\right))\cap \overset{K}{\underset{i=4}{⋂}}\nabla F{\left(\overset{¯}{x}\right)}^{T}Q_{i}^{\circ }=\ldots \\ & =\nabla F{\left(\overset{¯}{x}\right)}^{T}\left(Q_{1}^{\circ }\cap \overset{K}{\underset{i=2}{⋂}}\left(\ker\nabla F{\left(\overset{¯}{x}\right)}^{T}+Q_{i}^{\circ }\right)\right). \end{array}$$

Here, we use the convention that for sets $S_{1},\ldots ,S_{K}\subset R^{m}$ we set ${⋂}_{i=l}^{K}S_{i}=R^{m}$ for l>K. It is an easy consequence of (11), that equality holds in this inclusion, provided $\nabla F{\left(\overset{¯}{x}\right)}^{T}(Q_{1}^{\circ }\cap {⋂}_{i=2}^{K}\left(\ker\nabla F{\left(\overset{¯}{x}\right)}^{T}+Q_{i}^{\circ }\right))\subset \nabla F{\left(\overset{¯}{x}\right)}^{T}{\hat{N}}_{D}(F\left(\overset{¯}{x}\right)$. Hence, we have shown the following theorem.

Theorem 2: Assume that GGCQ holds at $\overset{¯}{x}\in \Omega$ and assume that $Q_{1},\ldots ,Q_{K}$ are convex cones contained in $T_{D}\left(F\left(\overset{¯}{x}\right)\right)$. If

(19) $$\begin{array}{c} {\left(\nabla F{\left(\overset{¯}{x}\right)}^{-1}Q_{i}\right)}^{\circ }=\nabla F{\left(\overset{¯}{x}\right)}^{T}Q_{i}^{\circ },\;i=1,\ldots ,K, \end{array}$$

then

(20) $$\begin{array}{c} {\hat{N}}_{\Omega }\left(\overset{¯}{x}\right)\subset \nabla F{\left(\overset{¯}{x}\right)}^{T}\left(Q_{1}^{\circ }\cap \overset{K}{\underset{i=2}{⋂}}\left(\ker\nabla F{\left(\overset{¯}{x}\right)}^{T}+Q_{i}^{\circ }\right)\right)=\overset{K}{\underset{i=1}{⋂}}\nabla F{\left(\overset{¯}{x}\right)}^{T}Q_{i}^{\circ }. \end{array}$$

Further, if

(21) $$\begin{array}{c} \nabla F{\left(\overset{¯}{x}\right)}^{T}\left(Q_{1}^{\circ }\cap \overset{K}{\underset{i=2}{⋂}}\left(\ker\nabla F{\left(\overset{¯}{x}\right)}^{T}+Q_{i}^{\circ }\right)\right)\subset \nabla F{\left(\overset{¯}{x}\right)}^{T}{\hat{N}}_{D}\left(F\left(\overset{¯}{x}\right)\right), \end{array}$$

then equality holds in (20) and ${\hat{N}}_{\Omega }\left(\overset{¯}{x}\right)=\nabla F{\left(\overset{¯}{x}\right)}^{T}{\hat{N}}_{D}\left(F\left(\overset{¯}{x}\right)\right)$.

Remark 1: Condition (19) is e.g. fulfilled, if for each i=1,…,K either there is a direction $u_{i}$ with $\nabla F\left(\overset{¯}{x}\right)u_{i}\in \mathrm{ri}\;Q_{i}$ or $Q_{i}$ is a convex polyhedral set, cf. [14, Proposition 1].

The proper choice of $Q_{1},\ldots ,Q_{K}$ is crucial in order that (20) provides a good estimate for the regular normal cone. It is obvious that we want to choose the cones $Q_{i}$, i=1,…,K as large as possible in order that the inclusion (20) is tight. Further, it is reasonable that a good choice of $Q_{1},\ldots ,Q_{K}$ fulfills

(22) $$\begin{array}{c} \overset{K}{\underset{i=1}{⋂}}Q_{i}^{\circ }={\hat{N}}_{D}\left(F\left(\overset{¯}{x}\right)\right) \end{array}$$

because then equation (21) holds whenever $\nabla F(\overset{¯}{x})$ has full rank. We now show that (21) holds not only under this full rank condition but also under some weaker assumption.

Theorem 3: Assume that GGCQ holds at $\overset{¯}{x}\in \Omega$ and assume that we are given convex cones $Q_{1},\ldots ,Q_{K}\subset T_{D}\left(F\left(\overset{¯}{x}\right)\right)$ fulfilling (19), (22) and

(23) $$\begin{array}{c} \ker\nabla F{\left(\overset{¯}{x}\right)}^{T}\cap \left(Q_{1}^{\circ }-Q_{i}^{\circ }\right)=\left\{0\right\},i=2,\ldots ,K. \end{array}$$

Then

$${\hat{N}}_{\Omega }\left(\overset{¯}{x}\right)=\nabla F{\left(\overset{¯}{x}\right)}^{T}{\hat{N}}_{D}\left(F\left(\overset{¯}{x}\right)\right)=\nabla F{\left(\overset{¯}{x}\right)}^{T}\left(Q_{1}^{\circ }\cap \overset{K}{\underset{i=2}{⋂}}\left(\ker\nabla F{\left(\overset{¯}{x}\right)}^{T}+Q_{i}^{\circ }\right)\right).$$

In particular, (23) holds if there is a subspace

(24) $$\begin{array}{c} L\subset \overset{K}{\underset{i=1}{⋂}}(Q_{i}\cap \left(-Q_{i}\right)) \end{array}$$

such that (13) holds.

**Proof **The statement follows from Theorem 2 if we can show that (21) holds. Consider $x^{*}\in \nabla F{\left(\overset{¯}{x}\right)}^{T}(Q_{1}^{\circ }\cap {⋂}_{i=2}^{K}\left(\ker\nabla F{\left(\overset{¯}{x}\right)}^{T}+Q_{i}^{\circ }\right))$. Then there are elements $\lambda^{i}\in Q_{i}^{\circ }$, i=1,…,K and $\mu^{i}\in \ker\nabla F{\left(\overset{¯}{x}\right)}^{T}$ such that $\lambda^{1}=\mu^{i}+\lambda^{i}$, i=2,…,K and $x^{*}=\nabla F{\left(\overset{¯}{x}\right)}^{T}\lambda^{1}$. We conclude $\mu^{i}=\lambda^{1}-\lambda^{i}\in Q_{1}^{\circ }-Q_{i}^{\circ }$, implying $\mu^{i}\in \ker\nabla F{\left(\overset{¯}{x}\right)}^{T}\cap \left(Q_{1}^{\circ }-Q_{i}^{\circ }\right)=\left\{0\right\}$ and thus

$$\begin{array}{c} \lambda^{1}=\lambda^{2}=\ldots =\lambda^{K}\in \overset{K}{\underset{i=1}{⋂}}Q_{i}^{\circ }={\hat{N}}_{D}\left(F\left(\overset{¯}{x}\right)\right). \end{array}$$

Hence, $x^{*}\in \nabla F{\left(\overset{¯}{x}\right)}^{T}{\hat{N}}_{D}\left(F\left(\overset{¯}{x}\right)\right)$ and (21) is verified. In order to show the last assertion note that from (24), we conclude $L\subset Q_{i}$ and consequently $Q_{i}^{\circ }\subset L^{\circ }=L^{⊥}$. Thus $Q_{1}^{\circ }-Q_{i}^{\circ }\subset L^{⊥}-L^{⊥}=L^{⊥}$, i=2,…,K. Since

$$\begin{array}{c} \ker\nabla F{\left(\overset{¯}{x}\right)}^{T}\cap L^{⊥}=({\left(\ker\nabla F{\left(\overset{¯}{x}\right)}^{T}\right)}^{⊥}+L{)}^{⊥}={\left(\nabla F\left(\overset{¯}{x}\right)R^{n}+L\right)}^{⊥}=\left\{0\right\}, \end{array}$$

it follows that (23) holds.

Corollary 1: Assume that GGCQ holds at $\overset{¯}{x}\in \Omega$ and assume that we are given convex cones $Q_{1},\ldots ,Q_{K}\subset T_{D}\left(F\left(\overset{¯}{x}\right)\right)$ fulfilling (19) and (22). Further assume that there is some subspace *L* fulfilling (12) and (13). Then, the sets

$$\begin{array}{c} {\tilde{Q}}_{i}:=Q_{i}+L,\;i=1,\ldots ,K \end{array}$$

are convex cones contained in $T_{D}\left(F\left(\overset{¯}{x}\right)\right)$,

(25) $$\begin{array}{c} {\left(\nabla F{\left(\overset{¯}{x}\right)}^{-1}{\tilde{Q}}_{i}\right)}^{\circ }=\nabla F{\left(\overset{¯}{x}\right)}^{T}{\tilde{Q}}_{i}^{\circ },\;i=1,\ldots ,K \end{array}$$

and

$${\hat{N}}_{\Omega }\left(\overset{¯}{x}\right)=\nabla F{\left(\overset{¯}{x}\right)}^{T}{\hat{N}}_{D}\left(F\left(\overset{¯}{x}\right)\right)=\nabla F{\left(\overset{¯}{x}\right)}^{T}\left({\tilde{Q}}_{1}^{\circ }\cap \overset{K}{\underset{i=2}{⋂}}\left(\ker\nabla F{\left(\overset{¯}{x}\right)}^{T}+{\tilde{Q}}_{i}^{\circ }\right)\right).$$

**Proof **Firstly observe that ${\tilde{Q}}_{i}=Q_{i}+L\subset T_{D}\left(F\left(\overset{¯}{x}\right)\right)+L\subset T_{D}\left(F\left(\overset{¯}{x}\right)\right)$ by (12). Next, consider $z\in \mathrm{ri}\;{\tilde{Q}}_{i}$. By (13) there exists $u\in R^{n}$ and l∈L such that $\nabla F(\overset{¯}{x})u+l=z$. Because of $-l\in L\subset {\tilde{Q}}_{i}$ we have $z-2l\in {\tilde{Q}}_{i}$ and thus $\nabla F\left(\overset{¯}{x}\right)u=z-l=\frac{1}{2}z+\frac{1}{2}\left(z-2l\right)\in \mathrm{ri}\;{\tilde{Q}}_{i}$ by [22, Theorem 6.1] implying (25) by taking into account Remark 1. Further, from $Q_{i}\subset {\tilde{Q}}_{i}\subset T_{D}\left(F\left(\overset{¯}{x}\right)\right)$ it follows that

$$\begin{array}{c} {\hat{N}}_{D}\left(F\left(\overset{¯}{x}\right)\right)={\left(T_{D}\left(F\left(\overset{¯}{x}\right)\right)\right)}^{\circ }\subset \overset{K}{\underset{i=1}{⋂}}{\tilde{Q}}_{i}^{\circ }\subset \overset{K}{\underset{i=1}{⋂}}Q_{i}^{\circ }={\hat{N}}_{D}\left(F\left(\overset{¯}{x}\right)\right). \end{array}$$

Finally, note that $L\subset {\tilde{Q}}_{i}\cap \left(-{\tilde{Q}}_{i}\right)$, i=1,…,K and the assertion follows from Theorem 3.

The following definition is motivated by Theorem 2.

Definition 5: Let $\overset{¯}{x}$ be feasible for the program (7) and let $Q_{1},\ldots ,Q_{K}$ be convex cones contained in $T_{D}\left(F\left(\overset{¯}{x}\right)\right)$ fulfilling (19).

(i) We say that $\overset{¯}{x}$ is Q-stationary with respect to $Q_{1},\ldots ,Q_{K}$, if
  $$\begin{array}{c} -\nabla f\left(\overset{¯}{x}\right)\in \nabla F{\left(\overset{¯}{x}\right)}^{T}\left(Q_{1}^{\circ }\cap \overset{K}{\underset{i=2}{⋂}}\left(\ker\nabla F{\left(\overset{¯}{x}\right)}^{T}+Q_{i}^{\circ }\right)\right). \end{array}$$
(ii) We say that $\overset{¯}{x}$ is $Q_{M}$-stationary with respect to $Q_{1},\ldots ,Q_{K}$, if
  $$\begin{array}{c} -\nabla f\left(\overset{¯}{x}\right)\in \nabla F{\left(\overset{¯}{x}\right)}^{T}\left(N_{D}\left(F\left(\overset{¯}{x}\right)\right)\cap Q_{1}^{\circ }\cap \overset{K}{\underset{i=2}{⋂}}\left(\ker\nabla F{\left(\overset{¯}{x}\right)}^{T}+Q_{i}^{\circ }\right)\right). \end{array}$$

Note that this definition is an extension of the definition of Q- and $Q_{M}$-stationarity in [14], where only the case K=2 was considered.

The following corollary is an immediate consequence of the definitions and Theorem 2.

Corollary 2: Assume that GGCQ is fulfilled at the point $\overset{¯}{x}$ feasible for (7). Further assume that we are given convex cones $Q_{1},\ldots ,Q_{K}\subset T_{D}\left(F\left(\overset{¯}{x}\right)\right)$ fulfilling (19). If $\overset{¯}{x}$ is B-stationary, then $\overset{¯}{x}$ is Q-stationary with respect to $Q_{1},\ldots ,Q_{K}$. Conversely, if $\overset{¯}{x}$ is Q-stationary with respect to $Q_{1},\ldots ,Q_{K}$ and (21) is fulfilled, then $\overset{¯}{x}$ is S-stationary and consequently B-stationary.

We know that under the assumptions of Proposition 1 every B-stationary point $\overset{¯}{x}$ for the problem (7) is both M-stationary and Q-stationary with respect to every collection of cones $Q_{1},\ldots ,Q_{K}\subset T_{D}\left(F\left(\overset{¯}{x}\right)\right)$ fulfilling (19), i.e.

$$\begin{array}{ccc} -\nabla f(\overset{¯}{x}) & \in & \nabla F{\left(\overset{¯}{x}\right)}^{T}N_{D}\left(F\left(\overset{¯}{x}\right)\right)\cap \nabla F{\left(\overset{¯}{x}\right)}^{T}\left(Q_{1}^{\circ }\cap \overset{K}{\underset{i=2}{⋂}}\left(\ker\nabla F{\left(\overset{¯}{x}\right)}^{T}+Q_{i}^{\circ }\right)\right)\\ & =\nabla F{\left(\overset{¯}{x}\right)}^{T}\left((\ker\nabla F{\left(\overset{¯}{x}\right)}^{T}+N_{D}\left(F\left(\overset{¯}{x}\right)\right))\cap Q_{1}^{\circ }\cap \overset{K}{\underset{i=2}{⋂}}\left(\ker\nabla F{\left(\overset{¯}{x}\right)}^{T}+Q_{i}^{\circ }\right)\right). \end{array}$$

Comparing this relation with the definition of $Q_{M}$-stationarity we see that $Q_{M}$-stationarity with respect to $Q_{1},\ldots ,Q_{K}$ is stronger than the simultaneous fulfilment of M-stationarity and Q-stationarity with respect to $Q_{1},\ldots ,Q_{K}$. We refer to [14, Example 2] for an example which shows that $Q_{M}$-stationarity is strictly stronger than M-stationarity. This is one of the advantages of $Q_{M}$-stationarity:

However, to ensure $Q_{M}$-stationarity of a B-stationary point $\overset{¯}{x}$, some additional assumption has to be fulfilled.

Lemma 1: Let $\overset{¯}{x}$ be B-stationary for the program (7) and assume that the assumptions of Proposition 1 are fulfilled at $\overset{¯}{x}$. Further assume that for every $\lambda \in N_{T_{D}\left(F\left(\overset{¯}{x}\right)\right)}\left(0\right)$ there exists a convex cone $Q_{\lambda }\subset T_{D}\left(F\left(\overset{¯}{x}\right)\right)$ containing λ and satisfying ${\left(\nabla F{\left(\overset{¯}{x}\right)}^{-1}Q_{\lambda }\right)}^{\circ }=\nabla F{\left(\overset{¯}{x}\right)}^{T}Q_{\lambda }^{\circ }$. Then there exists a convex cone $Q_{1}\subset T_{D}\left(F\left(\overset{¯}{x}\right)\right)$ fulfilling ${\left(\nabla F{\left(\overset{¯}{x}\right)}^{-1}Q_{1}\right)}^{\circ }=\nabla F{\left(\overset{¯}{x}\right)}^{T}Q_{1}^{\circ }$ such that for every collection $Q_{2},\ldots ,Q_{K}\subset T_{D}\left(F\left(\overset{¯}{x}\right)\right)$ fulfilling (19) the point $\overset{¯}{x}$ is $Q_{M}$-stationary with respect to $Q_{1},\ldots ,Q_{K}$.

**Proof **From the definition of B-stationarity and (16) we deduce the existence of $\lambda \in N_{T_{D}\left(F\left(\overset{¯}{x}\right)\right)}\left(0\right)$ fulfilling $-\nabla f\left(\overset{¯}{x}\right)=\nabla F{\left(\overset{¯}{x}\right)}^{T}\lambda$. By taking $Q_{1}=Q_{\lambda }$ we obviously have $\lambda \in N_{T_{D}\left(F\left(\overset{¯}{x}\right)\right)}\left(0\right)\cap Q_{1}^{\circ }\subset N_{D}\left(F\left(\overset{¯}{x}\right)\right)\cap Q_{1}^{\circ }$ implying $-\nabla f\left(\overset{¯}{x}\right)\in \nabla F{\left(\overset{¯}{x}\right)}^{T}\left(N_{D}\left(F\left(\overset{¯}{x}\right)\right)\cap Q_{1}^{\circ }\right)$. Now consider cones $Q_{2},\ldots ,Q_{K}\subset T_{D}\left(F\left(\overset{¯}{x}\right)\right)$ fulfilling (19). Similar to the derivation of Theorem 2 we obtain

$$\begin{array}{ccc} -\nabla f(\overset{¯}{x}) & \in & \nabla F{\left(\overset{¯}{x}\right)}^{T}\left(N_{D}\left(F\left(\overset{¯}{x}\right)\right)\cap Q_{1}^{\circ }\right)\cap \overset{K}{\underset{i=2}{⋂}}\nabla F{\left(\overset{¯}{x}\right)}^{T}Q_{i}^{\circ }\\ & =\nabla F{\left(\overset{¯}{x}\right)}^{T}\left(N_{D}\left(F\left(\overset{¯}{x}\right)\right)\cap Q_{1}^{\circ }\cap \overset{K}{\underset{i=2}{⋂}}\left(\ker\nabla F{\left(\overset{¯}{x}\right)}^{T}+Q_{i}^{\circ }\right)\right) \end{array}$$

and the lemma is proved.

Lemma 2: Let $\overset{¯}{x}$ be feasible for (7) and assume that $T_{D}\left(F\left(\overset{¯}{x}\right)\right)$ is the union of finitely many closed convex cones $C_{1},\ldots ,C_{p}$. Then for every $\lambda \in N_{T_{D}\left(F\left(\overset{¯}{x}\right)\right)}\left(0\right)$ there is some $\overset{¯}{i}\in \{1,\ldots ,p\}$ satisfying $\lambda \in C_{\overset{¯}{i}}^{\circ }$.

**Proof **Consider $\lambda \in N_{T_{D}\left(F\left(\overset{¯}{x}\right)\right)}\left(0\right)$. By the definition of the limiting normal cone there are sequences $t_{k}\overset{T_{D}\left(F\left(\overset{¯}{x}\right)\right)}{⟶}0$ and $\lambda_{k}\to \lambda$ with

$$\lambda_{k}\in {\hat{N}}_{T_{D}\left(F\left(\overset{¯}{x}\right)\right)}\left(t_{k}\right)={\left(\underset{i:t_{k}\in C_{i}}{⋃}T_{C_{i}}\left(t_{k}\right)\right)}^{\circ }=\underset{i:t_{k}\in C_{i}}{⋂}{\left(T_{C_{i}}\left(t_{k}\right)\right)}^{\circ }=\underset{i:t_{k}\in C_{i}}{⋂}N_{C_{i}}\left(t_{k}\right).$$

By passing to a subsequence if necessary we can assume that there is an index $\overset{¯}{i}$ such that $t_{k}\in C_{\overset{¯}{i}}$ for all *k* and we obtain $\lambda_{k}\in N_{C_{\overset{¯}{i}}}\left(t_{k}\right)=\left\{c^{*}\in C_{\overset{¯}{i}}^{\circ }\;|\;\left\langle c^{*},t_{k}\right\rangle =0\right\}\subset C_{\overset{¯}{i}}^{\circ }$. Since the polar cone $C_{\overset{¯}{i}}^{\circ }$ is closed, we deduce $\lambda \in C_{\overset{¯}{i}}^{\circ }$.

If $T_{D}\left(F\left(\overset{¯}{x}\right)\right)$ is the union of finitely many convex polyhedral cones $C_{1},\ldots ,C_{p}$, then the mapping $u⇉\nabla F\left(\overset{¯}{x}\right)u-T_{D}\left(F\left(\overset{¯}{x}\right)\right)$ is a polyhedral multifunction and thus metrically subregular at (0, 0) by Robinson’s result [23]. Further we know that for any convex polyhedral cone *Q* we have ${\left(\nabla F{\left(\overset{¯}{x}\right)}^{-1}Q\right)}^{\circ }=\nabla F{\left(\overset{¯}{x}\right)}^{T}Q^{\circ }$. Hence, we obtain the following corollary.

Corollary 3: Assume that $\overset{¯}{x}$ is B-stationary for the program (7), that GGCQ is fulfilled at $\overset{¯}{x}$ and that $T_{D}\left(F\left(\overset{¯}{x}\right)\right)$ is the union of finitely many convex polyhedral cones. Then there is a convex polyhedral cone $Q_{1}\subset T_{D}\left(F\left(\overset{¯}{x}\right)\right)$ such that for every collection $Q_{2},\ldots ,Q_{K}$ of convex polyhedral cones contained in $T_{D}\left(F\left(\overset{¯}{x}\right)\right)$ the point $\overset{¯}{x}$ is $Q_{M}$-stationary with respect to $Q_{1},\ldots ,Q_{K}$.

Let us notice that in contrast to S-, M- and many other types of stationarity the properties of Q- and $Q_{M}$-stationarity cannot be characterized by some single multiplier. In fact, *Q*- and $Q_{M}$-stationarity with respect to $Q_{1},\ldots ,Q_{K}$ implies the existence of *K* multipliers $\lambda_{1},\ldots ,\lambda_{K}$ satisfying

$$\begin{array}{c} \lambda_{i}\in Q_{i}^{\circ },\;\nabla f\left(\overset{¯}{x}\right)+\nabla F{\left(\overset{¯}{x}\right)}^{T}\lambda_{i}=0,\;i=1,\ldots ,K. \end{array}$$

In case of $Q_{M}$-stationarity the multiplier $\lambda_{1}$ also fulfills the M-stationarity conditions.

Further, let us note that $Q_{M}$-stationarity, although it is stronger that M-stationarity, does not imply B-stationarity in general. Thus, in general $Q_{M}$-stationarity is not a sufficient condition for a local minimizer as well.

## 4. Application to MPDC

It is clear that Q-stationarity is not a very strong optimality condition for every choice of $Q_{1},\ldots ,Q_{K}\subset T_{D}\left(F\left(\overset{¯}{x}\right)\right)$. As mentioned above the fulfillment of (22) is desirable. For the general problem (7), it can be impossible to choose the cones $Q_{1},\ldots ,Q_{K}$ such that (22) holds. If $T_{D}\left(F\left(\overset{¯}{x}\right)\right)$ is the union of finitely many convex cones $C_{1},\ldots ,C_{p}$ then we obviously have

$$\begin{array}{c} {\hat{N}}_{D}\left(F\left(\overset{¯}{x}\right)\right)=\overset{p}{\underset{i=1}{⋂}}C_{i}^{\circ }. \end{array}$$

However, to consider Q-stationarity with respect to $C_{1},\ldots ,C_{p}$ is in general not a feasible approach because *p* is often very large. We will now work out that the concepts of Q- and $Q_{M}$-stationarity are tailored for the MPDC (1). In what follows let *D* and *F* be given by (2).

Given a point $y=(y_{1},\ldots ,y_{m_{D}})\in D$, we denote by

$$\begin{array}{c} A_{i}\left(y\right):=\left\{j\in \left\{1,\ldots ,K_{i}\right\}\;|\;y_{i}\in D_{i}^{j}\right\},\;i=1,\ldots ,m_{D} \end{array}$$

the indices of sets $D_{i}^{j}$ which contain $y_{i}$. Further we choose for each $i=1,\ldots ,m_{D}$ some index set $J_{i}\left(y\right)\subset A_{i}\left(y\right)$ such that

(26) $$\begin{array}{c} T_{D_{i}}\left(y_{i}\right)=\underset{j\in J_{i}\left(y\right)}{⋃}T_{D_{i}^{j}}\left(y_{i}\right). \end{array}$$

Obviously the choice $J_{i}\left(y\right)=A_{i}\left(y\right)$ is feasible but for practical reasons it is better to choose $J_{i}\left(y\right)$ smaller if possible. E.g. if $T_{D_{i}^{j_{2}}}\left(y_{i}\right)\subset T_{D_{i}^{j_{1}}}\left(y_{i}\right)$ holds for some indices $j_{1},j_{2}\in A_{i}\left(y\right)$, then we will not include $j_{2}$ in $J_{i}\left(y\right)$. Such a situation can occur e.g. in case of MPVC when $(-H_{i}\left(\overset{¯}{x}\right),G_{i}\left(\overset{¯}{x}\right))=\left(0,a\right)$ with a<0.

Now consider

$$\begin{array}{c} \nu \in J\left(y\right):=\prod_{i=1}^{m_{D}}J_{i}\left(y\right). \end{array}$$

Since for every $i=1,\ldots ,m_{D}$ the set $D_{i}$ is the union of finitely many convex polyhedral sets, for every tangent direction $t\in T_{D_{i}}\left(y_{i}\right)$ we have $y_{i}+\alpha t\in D_{i}$ for all α>0 sufficiently small. Hence, we can apply [24, Proposition 1] to obtain

$$\begin{array}{c} T_{D(\nu )}\left(y\right)=\prod_{i=1}^{m_{D}}T_{D_{i}^{\nu_{i}}}\left(y_{i}\right),\;\nu \in J\left(y\right) \end{array}$$

with D(ν) given by (4), and

(27) $$\begin{array}{c} T_{D}\left(y\right)=\prod_{i=1}^{m_{D}}T_{D_{i}}\left(y_{i}\right)=\prod_{i=1}^{m_{D}}(\underset{j\in J_{i}\left(y\right)}{⋃}T_{D_{i}^{j}}\left(y_{i}\right))=\underset{\nu \in J(y)}{⋃}T_{D(\nu )}\left(y\right). \end{array}$$

We will apply this setting in particular to points $y=F(\overset{¯}{x})$ with $\overset{¯}{x}$ feasible for MPDC.

Lemma 3: Let $\overset{¯}{x}$ be feasible for the MPDC (1) and assume that we are given *K* elements $\nu^{1},\ldots ,\nu^{K}\in J\left(F\left(\overset{¯}{x}\right)\right)$ such that

(28) $$\begin{array}{c} \left\{\nu_{i}^{1},\ldots ,\nu_{i}^{K}\right\}=J_{i}\left(F\left(\overset{¯}{x}\right)\right),\;i=1,\ldots ,m_{D}. \end{array}$$

Then for each l=1,…,K the cone $Q_{l}:=T_{D(\nu^{l})}\left(F\left(\overset{¯}{x}\right)\right)$ is a convex polyhedral cone contained in $T_{D}\left(F\left(\overset{¯}{x}\right)\right)$, $(\nabla F{\left(\overset{¯}{x}\right)}^{-1}Q_{l}{)}^{\circ }=\nabla F{\left(\overset{¯}{x}\right)}^{T}Q_{l}^{\circ }$, and

$$\begin{array}{c} \overset{K}{\underset{l=1}{⋂}}Q_{l}^{\circ }={\hat{N}}_{D}\left(F\left(\overset{¯}{x}\right)\right). \end{array}$$

**Proof **Obviously, for every l=1,…,K the cone $Q_{l}$ is convex and polyhedral because it is the product of convex polyhedral cones. This implies $(\nabla F{\left(\overset{¯}{x}\right)}^{-1}Q_{l}{)}^{\circ }=\nabla F{\left(\overset{¯}{x}\right)}^{T}Q_{l}^{\circ }$ and $Q_{l}\subset T_{D}\left(F\left(\overset{¯}{x}\right)\right)$ follows from (27). By taking into account (27) the last assertion follows from

$$\begin{array}{cc} {\hat{N}}_{D}\left(F\left(\overset{¯}{x}\right)\right) & =(T_{D}\left(F\left(\overset{¯}{x}\right)\right){)}^{\circ }=\prod_{i=1}^{m_{D}}(\underset{j\in J_{i}\left(F\left(\overset{¯}{x}\right)\right)}{⋃}T_{D_{i}^{j}}\left(F_{i}\left(\overset{¯}{x}\right)\right){)}^{\circ }=\prod_{i=1}^{m_{D}}(\overset{K}{\underset{l=1}{⋃}}T_{D_{i}^{\nu_{i}^{l}}}\left(F_{i}\left(\overset{¯}{x}\right)\right){)}^{\circ }\\ & =\prod_{i=1}^{m_{D}}(\overset{K}{\underset{l=1}{⋂}}(T_{D_{i}^{\nu_{i}^{l}}}\left(F_{i}\left(\overset{¯}{x}\right)\right){)}^{\circ })=\overset{K}{\underset{l=1}{⋂}}(\prod_{i=1}^{m_{D}}(T_{D_{i}^{\nu_{i}^{l}}}\left(F_{i}\left(\overset{¯}{x}\right)\right){)}^{\circ })=\overset{K}{\underset{l=1}{⋂}}(\prod_{i=1}^{m_{D}}T_{D_{i}^{\nu_{i}^{l}}}\left(F_{i}\left(\overset{¯}{x}\right)\right){)}^{\circ }\\ & =\overset{K}{\underset{l=1}{⋂}}Q_{l}^{\circ }. \end{array}$$

Definition 6: Let $\overset{¯}{x}$ be feasible for the MPDC (1) and let index sets $J_{i}\left(F\left(\overset{¯}{x}\right)\right)\subset A_{i}\left(\overset{¯}{x}\right)$, $i=1,\ldots ,m_{D}$ fulfilling (26) be given. Further we denote by $Q(\overset{¯}{x})$ the collection of all elements $\left(\nu^{1},\ldots ,\nu^{K}\right)$ with $\nu^{l}\in J\left(F\left(\overset{¯}{x}\right)\right)=\prod_{i=1}^{m_{D}}J_{i}\left(F\left(\overset{¯}{x}\right)\right)$, l=1,…,K such that (28) holds.

(1) We say that $\overset{¯}{x}$ is Q-stationary ($Q_{M}$-stationary) for (1) with respect to $\left(\nu^{1},\ldots ,\nu^{K}\right)\in Q\left(\overset{¯}{x}\right)$, if $\overset{¯}{x}$ is Q-stationary ($Q_{M}$-stationary) with respect to $Q_{1},\ldots ,Q_{K}$ in the sense of Definition 5 with $Q_{l}:=T_{D(\nu^{l})}\left(F\left(\overset{¯}{x}\right)\right)$, l=1,…,K.
(2) We say that $\overset{¯}{x}$ is Q-stationary ($Q_{M}$-stationary) for (1) if $\overset{¯}{x}$ is Q-stationary ($Q_{M}$-stationary) for (1) with respect to some $\left(\nu^{1},\ldots ,\nu^{K}\right)\in Q\left(\overset{¯}{x}\right)$.

Definition 6 is an extension of the definition of Q- and $Q_{M}$-stationarity made for MPCC and MPVC in [14]. Note that the number *K* appearing in the definition of $Q(\overset{¯}{x})$ is not fixed. Denoting $K_{\min}\left(\overset{¯}{x}\right)$ the minimal number *K* such that $\left(\nu^{1},\ldots ,\nu^{K}\right)\in Q\left(\overset{¯}{x}\right)$, we obviously have

(29) $$\begin{array}{c} K_{\min}\left(\overset{¯}{x}\right)=\max_{i=1,\ldots ,m_{D}}\left|J_{i}\left(F\left(\overset{¯}{x}\right)\right)\right|\leq \max_{i=1,\ldots ,m_{D}}K_{i}. \end{array}$$

We see from (27) that the tangent cone $T_{D}\left(F\left(\overset{¯}{x}\right)\right)$ is the union of the $\left|J\left(F\left(\overset{¯}{x}\right)\right)\right|=\prod_{i=1}^{m_{D}}\left|J_{i}\left(F\left(\overset{¯}{x}\right)\right)\right|$ convex polyhedral cones $T_{D(\nu )}\left(y\right)$. Hence, the minimal number $K_{\min}\left(\overset{¯}{x}\right)$ is much smaller than the number of components of the tangent cone, except when all or nearly all sets $J_{i}\left(F\left(\overset{¯}{x}\right)\right)$ have cardinality 1. E.g. when $K_{i}\leq 2$ holds for all $i=1,\ldots ,m_{D}$ as it is the case of the MPCC (5), then we have $K_{\min}\left(\overset{¯}{x}\right)\leq 2$ whereas the number of convex cones building the tangent cone $T_{D}\left(F\left(\overset{¯}{x}\right)\right)$ grows exponentially with the number of biactive constraints, i.e. complementarity constraints satisfying $G_{i}\left(\overset{¯}{x}\right)=H_{i}\left(\overset{¯}{x}\right)=0$. The concepts of Q- and $Q_{M}$-stationarity for MPDC take advantage of the fact that although the tangent cone is the union of a huge number of cones, its polar cone can be written as the intersection of a small number of polars. Further, it is clear that for every $\nu^{1}\in J\left(F\left(\overset{¯}{x}\right)\right)$ and every $K\geq K_{\min}\left(\overset{¯}{x}\right)$ we can find $\nu^{2},\ldots ,\nu^{K}\in J\left(F\left(\overset{¯}{x}\right)\right)$ such that $\left(\nu^{1},\ldots ,\nu^{K}\right)\in Q\left(\overset{¯}{x}\right)$.

We allow *K* to be greater than $K_{\min}\left(\overset{¯}{x}\right)$ for numerical reasons primarily. Recall that for testing Q-stationarity with respect to $\left(\nu^{1},\ldots ,\nu^{K}\right)$, we have to check for all l=1,…,K whether $-\nabla f\left(\overset{¯}{x}\right)\in \nabla F{\left(\overset{¯}{x}\right)}^{T}Q_{l}^{\circ }$, or equivalently, that u=0 is a solution of the linear optimization program

$$\begin{array}{c} \min\left\langle \nabla f\left(\overset{¯}{x}\right),u\right\rangle \;\;\text{subject to}\;\nabla F\left(\overset{¯}{x}\right)u\in Q_{l} \end{array}$$

with $Q_{l}=T_{D(\nu^{l})}\left(F\left(\overset{¯}{x}\right)\right)$. Theoretically the treatment of degenerated linear constraints is not a big problem but the numerical practice tells us the contrary. In [25] we have implemented an algorithm for solving MPVC based on Q-stationarity and the degeneracy of the linear constraints was the reason when the algorithm crashed. The possibility of choosing $K>K_{\min}\left(\overset{¯}{x}\right)$ gives us more flexibility to avoid linear programs with degenerated constraints.

The following theorem follows from Corollaries 2, 3, Theorem 3 and the considerations above.

Theorem 4: Let $\overset{¯}{x}$ be feasible for the MPDC (1) and assume that GGCQ is fulfilled at $\overset{¯}{x}$.

(i) If $\overset{¯}{x}$ is B-stationary then $\overset{¯}{x}$ is Q-stationary with respect to every element $\left(\nu^{1},\ldots ,\nu^{K}\right)\in Q\left(\overset{¯}{x}\right)$ and there exists some ${\overset{¯}{\nu }}^{1}\in J\left(F\left(\overset{¯}{x}\right)\right)$ such that $\overset{¯}{x}$ is $Q_{M}$-stationary with respect to every $\left({\overset{¯}{\nu }}^{1},\nu^{2},\ldots ,\nu^{K}\right)\in Q\left(\overset{¯}{x}\right)$.
(ii) Conversely, if $\overset{¯}{x}$ is Q-stationary with respect to some $\left(\nu^{1},\ldots ,\nu^{K}\right)\in Q\left(\overset{¯}{x}\right)$ and
  (30) $$\begin{array}{c} \nabla F{\left(\overset{¯}{x}\right)}^{T}(Q_{1}^{\circ }\cap \overset{K}{\underset{l=2}{⋂}}\left(\ker\nabla F{\left(\overset{¯}{x}\right)}^{T}+Q_{l}^{\circ }\right))\subset \nabla F{\left(\overset{¯}{x}\right)}^{T}{\hat{N}}_{D}\left(F\left(\overset{¯}{x}\right)\right), \end{array}$$
  where $Q_{l}:=T_{D(\nu^{l})}F\left(\overset{¯}{x}\right)$, l=1,…,K, then $\overset{¯}{x}$ is S-stationary and consequently B-stationary. In particular, (30) is fulfilled if
  (31) $$\begin{array}{c} \ker\nabla F{\left(\overset{¯}{x}\right)}^{T}\cap (Q_{1}^{\circ }-Q_{l}^{\circ })=\left\{0\right\},\;l=2,\ldots ,K. \end{array}$$

## 5. On quadratic programs with disjunctive constraints

In this section, we consider the special case of quadratic programs with disjunctive constraints (QPDC)

(32) $$\begin{array}{cc} \min_{x\in R^{n}} & q\left(x\right):=\frac{1}{2}x^{T}Bx+d^{T}x\\ \;\text{subject to} & A_{i}x\in D_{i}:=\overset{K_{i}}{\underset{j=1}{⋃}}D_{i}^{j},\;i=1,\ldots ,m_{D}, \end{array}$$

where *B* is a positive semidefinite n×n matrix, $d\in R^{n}$, $A_{i}$, $i=1,\ldots ,m_{D}$ are $l_{i}\times n$ matrices and $D_{i}^{j}\subset R^{l_{i}}$, $i=1,\ldots ,m_{D}$, $j=1,\ldots ,K_{j}$ are convex polyhedral sets, i.e. QPDC is a special case of MPDC with f(x)=q(x) and $F_{i}\left(x\right)=A_{i}x$, $i=1,\ldots ,m_{D}$. In what follows, we denote by *A* the m×n matrix

$$\begin{array}{c} A=\left(\begin{array}{c} A_{1}\\ \vdots \\ A_{m_{D}} \end{array}\right), \end{array}$$

where $m:=\sum_{i=1}^{m_{D}}l_{i}$.

We start our analysis with the following preparatory lemma.

Lemma 4: Assume that the convex quadratic program

(33) $$\begin{array}{c} \min_{x\in R^{n}}\frac{1}{2}x^{T}Bx+d^{T}x\;\;\text{subject to}\;Ax\in C \end{array}$$

is feasible, where *B* is some symmetric positive semidefinite n×n matrix, $d\in R^{n}$, *A* is a m×n matrix and $C\subset R^{m}$ is a convex polyhedral set. Then either there exists a direction *w* satisfying

(34) $$\begin{array}{c} Bw=0,\;Aw\in 0^{+}C,\;d^{T}w<0, \end{array}$$

or the program (33) has a global solution $\overset{¯}{x}$.

**Proof **Assume that for every *w* with Bw=0, $Aw\in 0^{+}C$ we have $d^{T}w\geq 0$, i.e. 0 is a global solution of the program

$$\begin{array}{c} \min d^{T}w\;\;\text{subject to}\;w\in S:=\left\{w\;|\;\left(\begin{array}{c} B\\ A \end{array}\right)w\in {\left\{0\right\}}^{n}\times 0^{+}C\right\}. \end{array}$$

Since *C* is a convex polyhedral set, its recession cone $0^{+}C$ is a convex polyhedral cone and so is ${\left\{0\right\}}^{n}\times 0^{+}C$ as well. Hence,

$$\begin{array}{c} {\hat{N}}_{S}\left(0\right)=S^{\circ }=\left(B^{T}\vdots A^{T}\right){\left({\left\{0\right\}}^{n}\times 0^{+}C\right)}^{\circ }=B^{T}R^{n}+A^{T}{\left(0^{+}C\right)}^{\circ } \end{array}$$

and from the first-order optimality condition $-d\in {\hat{N}}_{S}\left(0\right)$ we derive the existence of multipliers $\mu_{B}\in R^{n}$ and $\mu_{C}\in {\left(0^{+}C\right)}^{\circ }$ such that

$$\begin{array}{c} -d=B^{T}\mu_{B}+A^{T}\mu_{C}. \end{array}$$

The convex polyhedral set *C* is the sum of the convex hull Σ of its extreme points and its recession cone. Hence, for every *x* feasible for (33) there is some $c_{1}\in \Sigma$ and some $c_{2}\in 0^{+}C$ such that $Ax=c_{1}+c_{2}$ and, by taking into account $\mu_{C}^{T}c_{2}\leq 0$, we obtain

(35) $$\begin{array}{ccc} \frac{1}{2}x^{T}Bx+d^{T}x & =\frac{1}{2}x^{T}Bx-\mu_{B}^{T}Bx-\mu_{C}^{T}Ax\\ & =\frac{1}{2}{\left(x-\mu_{B}\right)}^{T}B\left(x-\mu_{B}\right)-\frac{1}{2}\mu_{B}^{T}B\mu_{B}-\mu_{C}^{T}c_{1}-\mu_{C}^{T}c_{2}\\ & \geq & -\frac{1}{2}\mu_{B}^{T}B\mu_{B}-\mu_{C}^{T}c_{1}. \end{array}$$

The set Σ is compact and we conclude that the objective of (33) is bounded below on the feasible domain $A^{-1}C$ by $-\frac{1}{2}\mu_{B}^{T}B\mu_{B}-\max_{c_{1}\in \Sigma }\mu_{C}^{T}c_{1}$. Thus

$$\begin{array}{c} \alpha :=\inf\left\{\frac{1}{2}x^{T}Bx+d^{T}x\;|\;Ax\in C\right\} \end{array}$$

is finite and there remains to show that the infimum is attained. Consider some sequence $x_{k}\in A^{-1}C$ with $\lim_{k\to \infty }\frac{1}{2}x_{k}^{T}Bx_{k}+d^{T}x_{k}=\alpha$. We conclude from (35) that ${\left(x_{k}-\mu_{B}\right)}^{T}B\left(x_{k}-\mu_{B}\right)$ is bounded which in turn implies that the sequence $B^{1/2}x_{k}$ is bounded. Hence, the sequence $x_{k}^{T}Bx_{k}={\left\|B^{1/2}x_{k}\right\|}^{2}$ is bounded as well and we can conclude also the boundedness of $d^{T}x_{k}$. By passing to a subsequence we can assume that the sequence $\left(B^{1/2}x_{k},d^{T}x_{k}\right)$ converges to some (z,β) and it follows that $\alpha =\frac{1}{2}{\left\|z\right\|}^{2}+\beta$. Since *C* is a convex polyhedral set, it follows by applying [22, Theorem 19.3] twice, that the sets $A^{-1}C$ and $\left\{\left(B^{1/2}u,d^{T}u\right)\;|\;u\in A^{-1}C\right\}$ are convex and polyhedral. Since convex polyhedral sets are closed, it follows that $\left(z,\beta \right)\in \{\left(B^{1/2}u,d^{T}u\right)\;|\;u\in A^{-1}C\}$. Thus there is some $\overset{¯}{x}\in A^{-1}C$ with $\left(z,\beta \right)=\left(B^{1/2}\overset{¯}{x},d^{T}\overset{¯}{x}\right)$ and $\frac{1}{2}{\overset{¯}{x}}^{T}B\overset{¯}{x}+d^{T}\overset{¯}{x}=\alpha$ follows. This shows that $\overset{¯}{x}$ is a global minimizer for (33).

In what follows, we assume that we have at hand an algorithm for solving (33), which either computes a global solution $\overset{¯}{x}$ or a descent direction *w* fulfilling (34). Such an algorithm is, e.g. the active set method as described in [26], where we have to rewrite the constraints equivalently in the form $\left\langle A^{T}a_{i},x\right\rangle \leq b_{i}$, i=1,…,p using the representation of *C* as the intersection of finitely many half-spaces, $C=\{c\;|\;\left\langle a_{i},c\right\rangle \leq b_{i},\;i=1,\ldots ,p\}$.

Consider now the following algorithm. 
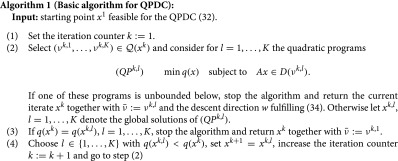


Algorithm 1 can be considered as a kind of active index set strategy. The set $\left(\nu^{k,1},\ldots ,\nu^{k,K}\right)\in Q\left(x^{k}\right)$ chosen in step (2) acts as a working set and is a subset of the active pieces of the disjunctive constraints. The number *K* will also depend on $x^{k}$ and for practical reasons it is desirable to keep *K* small to have small numerical effort in each iteration. Recall that we can always choose *K* equal to the number $K_{\min}\left(x^{k}\right)$ given by (29) which is bounded by $\max_{i=1,\ldots ,m_{D}}K_{i}$. The working set is used for testing for unboundedness of the problem and Q-stationarity, respectively, by investigating the quadratic subproblems $\left(QP^{k,l}\right)$. If one of these subproblem’s problem appears unbounded, we stop the algorithm because of unboundedness of the whole program. If $x^{k}$ is a solution for every quadratic subproblem, we stop the algorithm because $x^{k}$ is Q-stationary. On the other hand, if $x^{k}$ is not a solution of one of these subproblems, then we take the point $x^{k+1}$ as the solution of this subproblem, yielding a smaller objective function value. Then, we repeat the whole procedure by generating a new working set and testing for termination.

In the next theorem, we show that Algorithm 1 is finite. However, we do not know any nontrivial bound on the number of iterations needed, as usual for active set strategies.

Theorem 5: Algorithm 1 terminates after a finite number of iterations either with some feasible point and some descent direction *w* indicating that QPDC is unbounded below or with some Q-stationary solution.

**Proof **If Algorithm 1 terminates in step (2) the output is a feasible point together with some descent direction showing that QPDC is unbounded below. If the algorithm does not terminate in step (2), the computed sequence of function values $q(x^{k})$ is strictly decreasing. Moreover, denoting $\nu^{k}:=\nu^{k-1,l}$ where *l* is the index chosen in step (4), we see that for each k≥2 the point $x^{k}$ is global minimizer of the problem

$$\begin{array}{c} \;\min q\left(x\right)\;\;\text{subject to}\;Ax\in D\left(\nu^{k}\right). \end{array}$$

This shows that all the vectors $\nu^{k}$ must be pairwise different and since there is only a finite number of possible choices for $\nu^{k}$, the algorithm must stop in step (3). We will now show that the final iterate $x^{k}$ is Q-stationary with respect to $\left(\nu^{k,1},\ldots ,\nu^{k,K}\right)$. Since for each l=1,…,K the point $x^{k}$ is a global minimizer of the subproblem $\left(Q^{k,l}\right)$, it also satisfies the first order optimality condition

$$\begin{array}{c} \left\langle \nabla q\left(x^{k}\right),u\right\rangle \geq 0\;\;\text{for every}u\in R^{n}\;\text{satisfying}\;Au\in T_{D(\nu^{k,l})}\left(Ax^{k}\right)). \end{array}$$

This shows Q-stationarity of $x^{k}$ and the theorem is proved.

## On verifying $Q_{M}$-stationarity for MPDC

6.

The following theorem is crucial for the verification of M-stationarity.

Theorem 6:

(i) Let $\overset{¯}{x}$ be feasible for the general program (7). If there exists a B-stationary solution of the program
  (36) $$\begin{array}{c} \min_{\left(u,v\right)\in R^{n}\times R^{m}}\left\langle \nabla f\left(\overset{¯}{x}\right),u\right\rangle +\frac{1}{2}{\left\|v\right\|}^{2}\;\;\text{subject to}\;\nabla F\left(\overset{¯}{x}\right)u+v\in T_{D}\left(F\left(\overset{¯}{x}\right)\right), \end{array}$$
  then $\overset{¯}{x}$ is M-stationary.
(ii) Let $\overset{¯}{x}$ be B-stationary for the MPDC (1) and assume that GGCQ holds at $\overset{¯}{x}$. Then the program (36) has a global solution.

**Proof**

(i) Let $\left(\overset{¯}{u},\overset{¯}{v}\right)$ denote a B-stationary solution, i.e. $-\left(\nabla f\left(\overset{¯}{x}\right),\overset{¯}{v}\right)\in {\hat{N}}_{\Gamma }\left(\overset{¯}{u},\overset{¯}{v}\right)$, where $\Gamma ={\left(\nabla F\left(\overset{¯}{x}\right)\;\vdots \;I\right)}^{-1}T_{D}\left(F\left(\overset{¯}{x}\right)\right)$. Since the matrix $\left(\nabla F(\overset{¯}{x})\;\vdots \;I\right)$ obviously has full rank, we have ${\hat{N}}_{\Gamma }\left(\overset{¯}{u},\overset{¯}{v}\right)={\left(\nabla F\left(\overset{¯}{x}\right)\;\vdots \;I\right)}^{T}{\hat{N}}_{T_{D}\left(F\left(\overset{¯}{x}\right)\right)}\left(\nabla F\left(\overset{¯}{x}\right)\overset{¯}{u}+\overset{¯}{v}\right)$ by [11, Exercise 6.7]. Thus there exists a multiplier $\lambda \in {\hat{N}}_{T_{D}\left(F\left(\overset{¯}{x}\right)\right)}\left(\nabla F\left(\overset{¯}{x}\right)\overset{¯}{u}+\overset{¯}{v}\right)$ such that $-\nabla f\left(\overset{¯}{x}\right)=\nabla F{\left(\overset{¯}{x}\right)}^{T}\lambda$ and $-\overset{¯}{v}=\lambda$. Using [11, Proposition 6.27] we have ${\hat{N}}_{T_{D}\left(F\left(\overset{¯}{x}\right)\right)}\left(\nabla F\left(\overset{¯}{x}\right)\overset{¯}{u}+\overset{¯}{v}\right)\subset N_{T_{D}\left(F\left(\overset{¯}{x}\right)\right)}\left(\nabla F\left(\overset{¯}{x}\right)\overset{¯}{u}+\overset{¯}{v}\right)\subset N_{T_{D}\left(F\left(\overset{¯}{x}\right)\right)}\left(0\right)\subset N_{D}\left(F\left(\overset{¯}{x}\right)\right)$ establishing M-stationarity of $\overset{¯}{x}$.
(ii) Consider for arbitrarily fixed $\nu \in J(F(\overset{¯}{x}))$ the convex quadratic program
  (37) $$\begin{array}{c} \min_{\left(u,v\right)\in R^{n}\times R^{m}}\left\langle \nabla f\left(\overset{¯}{x}\right),u\right\rangle +\frac{1}{2}{\left\|v\right\|}^{2}\;\;\text{subject to}\;\nabla F\left(\overset{¯}{x}\right)u+v\in T_{D(\nu )}\left(F\left(\overset{¯}{x}\right)\right). \end{array}$$
  Assuming that this quadratic program does not have a solution, by Lemma 4 we could find a direction $\left(w_{u},w_{v}\right)$ satisfying
  $$\begin{array}{c} \left(\begin{array}{cc} 0 & 0\\ 0 & I \end{array}\right)\left(\begin{array}{c} w_{u}\\ w_{v} \end{array}\right)=0,\;\nabla F\left(\overset{¯}{x}\right)w_{u}+w_{v}\in 0^{+}T_{D(\nu )}\left(F\left(\overset{¯}{x}\right)\right),\;\left\langle \nabla f\left(\overset{¯}{x}\right),w_{u}\right\rangle +\left\langle 0,w_{v}\right\rangle <0. \end{array}$$
  This implies $w_{v}=0$, $\nabla F\left(\overset{¯}{x}\right)w_{u}\in 0^{+}T_{D(\nu )}\left(F\left(\overset{¯}{x}\right)\right)=T_{D(\nu )}\left(F\left(\overset{¯}{x}\right)\right)\subset T_{D}\left(F\left(\overset{¯}{x}\right)\right)$ and $\langle \nabla f\left(\overset{¯}{x}\right),w_{u}\rangle <0$ and thus, together with GGCQ, $-\nabla f\left(\overset{¯}{x}\right)\notin {\left(T_{\Omega }^{\mathrm{lin}}\left(\overset{¯}{x}\right)\right)}^{\circ }={\hat{N}}_{D}\left(F\left(\overset{¯}{x}\right)\right)$ contradicting our assumption that $\overset{¯}{x}$ is B-stationary for (1). Hence, the quadratic program (37) must possess some global solution $\left(u_{\nu },v_{\nu }\right)$. By choosing $\overset{¯}{\nu }\in J(F(\overset{¯}{x}))$ such that $\left\langle \nabla f\left(\overset{¯}{x}\right),u_{\overset{¯}{\nu }}\right\rangle =\min\left\{\left\langle \nabla f\left(\overset{¯}{x}\right),u_{\nu }\right\rangle \;|\;\nu \in J\left(F\left(\overset{¯}{x}\right)\right)\right\}$ it follows from (27) that $\left(u_{\overset{¯}{\nu }},v_{\overset{¯}{\nu }}\right)$ is a global solution of (36).

We now want to apply Algorithm 1 to the problem (36). Note that the point (0, 0) is feasible for (36) and therefore we can start Algorithm 1 with $\left(u^{1},v^{1}\right)=\left(0,0\right)$.

Corollary 4: Let $\overset{¯}{x}$ be feasible for the MPDC (1) and apply Algorithm 1 to the QPDC (36). If the algorithm returns an iterate together with some descent direction indicating that (36) is unbounded below and if GGCQ is fulfilled at $\overset{¯}{x}$, then $\overset{¯}{x}$ is not B-stationary. On the other hand, if the algorithm returns a Q-stationary solution, then $\overset{¯}{x}$ is M-stationary.

**Proof **Observe that in case when Algorithm 1 returns a Q-stationary solution, by Theorem 4(ii) this solution is B-stationary because the Jocobian of the constraints $\left(\nabla F(\overset{¯}{x})\;\vdots \;I\right)$ obviously has full rank. Now the statement follows from Theorem 6.

We now want to analyse how the output of Algorithm 1 can be further utilized. Recalling that $T_{D}\left(F\left(\overset{¯}{x}\right)\right)$ has the disjunctive structure

$$\begin{array}{c} T_{D}\left(F\left(\overset{¯}{x}\right)\right)=\prod_{i=1}^{m_{D}}(\underset{j\in J_{i}\left(F\left(\overset{¯}{x}\right)\right)}{⋃}T_{D_{i}^{j}}\left(F_{i}\left(\overset{¯}{x}\right)\right)), \end{array}$$

we define for $y=\left(y_{1},\ldots ,y_{m_{D}}\right)\in T_{D}\left(F\left(\overset{¯}{x}\right)\right)$ the index sets

$$\begin{array}{c} A_{i}^{T_{D}}\left(y\right):=\left\{j\in J_{i}\left(F\left(\overset{¯}{x}\right)\right)\;|\;y_{i}\in T_{D_{i}^{j}}\left(F_{i}\left(\overset{¯}{x}\right)\right)\right\},\;i=1,\ldots ,m_{D}. \end{array}$$

Further we choose for each $i=1,\ldots ,m_{D}$ some index set $J_{i}^{T_{D}}\left(y\right)\subset A_{i}^{T_{D}}\left(y\right)$ such that

(38) $$\begin{array}{c} T_{T_{D_{i}}\left(F_{i}\left(\overset{¯}{x}\right)\right)}\left(y_{i}\right)=\underset{j\in J_{i}^{T_{D}}\left(y\right)}{⋃}T_{T_{D_{i}^{j}}\left(F_{i}\left(\overset{¯}{x}\right)\right)}\left(y_{i}\right) \end{array}$$

and set

$$\begin{array}{c} J^{T_{D}}\left(y\right):=\prod_{i=1}^{m_{D}}J_{i}^{T_{D}}\left(y\right). \end{array}$$

Note that we always have

$$\begin{array}{c} J^{T_{D}}\left(y\right)\subset J\left(F\left(\overset{¯}{x}\right)\right). \end{array}$$

In order to verify Q-stationarity for the problem (36) at some feasible point (*u*, *v*), we have to consider the set $Q^{T_{D}}\left(u,v\right)$ consisting of all $\left(\nu^{1},\ldots ,\nu^{K}\right)$ with $\nu^{l}\in J^{T_{D}}\left(\nabla F\left(\overset{¯}{x}\right)u+v\right)$, l=1,…,K such that

$$\begin{array}{c} \left\{\nu_{i}^{1},\ldots ,\nu_{i}^{K}\right\}=J_{i}^{T_{D}}\left(\nabla F\left(\overset{¯}{x}\right)u+v\right),\;i=1,\ldots ,m_{D}. \end{array}$$

At the *k*-th iterate $\left(u^{k},v^{k}\right)$ we have to choose $\left(\nu^{k,1},\ldots ,\nu^{k,K}\right)\in Q^{T_{D}}\left(u^{k},v^{k}\right)$ and then for each l=1,…,K we must analyse the convex quadratic program

$$\begin{array}{c} \left(QP^{k,l}\right)\;\min_{u,v}\left\langle \nabla f\left(\overset{¯}{x}\right),u\right\rangle +\frac{1}{2}{\left\|v\right\|}^{2}\;\;\text{subject to}\;\nabla F\left(\overset{¯}{x}\right)u+v\in T_{D(\nu^{k,l})}\left(F\left(\overset{¯}{x}\right)\right). \end{array}$$

If for some $\overset{¯}{l}\in \{1,\ldots ,K\}$ this quadratic program is unbounded below then Algorithm 1 returns the index $\overset{¯}{\nu }:=\nu^{k,\overset{¯}{l}}$ together with a descent direction $\left(w_{u},w_{v}\right)$ fulfilling, as argued in the proof of Theorem 6(ii),

$$\begin{array}{c} w_{v}=0,\;\nabla F\left(\overset{¯}{x}\right)w_{u}\in 0^{+}T_{D(\overset{¯}{\nu })}\left(F\left(\overset{¯}{x}\right)\right)=T_{D(\overset{¯}{\nu })}\left(F\left(\overset{¯}{x}\right)\right),\left\langle \nabla f\left(\overset{¯}{x}\right),w_{u}\right\rangle <0. \end{array}$$

Therefore, $w_{u}$ constitutes a feasible descent direction, provided GACQ holds at $\overset{¯}{x}$, i.e. for every α>0 sufficiently small the projection of $\overset{¯}{x}+\alpha w_{u}$ on the feasible set $F^{-1}\left(D\right)$ yields a point with a smaller objective function value than $\overset{¯}{x}$. If GACQ also holds for the constraint $F(x)\in D(\overset{¯}{\nu })$ at $\overset{¯}{x}$, then we can also project the point $\overset{¯}{x}+\alpha w_{u}$ on $F^{-1}\left(D\left(\overset{¯}{\nu }\right)\right)$ in order to reduce the objective function.

Now, assume that the final iterate $\left(u^{k},v^{k}\right)$ of Algorithm 1 is Q-stationary for (36) and consequently $\overset{¯}{x}$ is M-stationary for the MPDC (1). Setting $\lambda :=-v^{k}$, the first order optimality conditions for the quadratic programs $\left(QP^{k,l}\right)$ result in

$$\begin{array}{ccc} -\nabla f(\overset{¯}{x}) & =\nabla F{\left(\overset{¯}{x}\right)}^{T}\lambda ,\\ \lambda & \in & \overset{K}{\underset{l=1}{⋂}}N_{T_{D(\nu^{k,l})}\left(F\left(\overset{¯}{x}\right)\right)}\left(\nabla F\left(\overset{¯}{x}\right)u^{k}+v^{k}\right)={\hat{N}}_{T_{D}\left(F\left(\overset{¯}{x}\right)\right)}\left(\nabla F\left(\overset{¯}{x}\right)u^{k}+v^{k}\right)\subset N_{D}\left(F\left(\overset{¯}{x}\right)\right). \end{array}$$

From this we conclude $-\nabla f\left(\overset{¯}{x}\right)\in \nabla F{\left(\overset{¯}{x}\right)}^{T}(Q_{1}^{\circ }\cap N_{D}\left(F\left(\overset{¯}{x}\right)\right)$ with $Q_{1}:=T_{D(\overset{¯}{\nu })}\left(F\left(\overset{¯}{x}\right)\right)\subset T_{T_{D(\overset{¯}{\nu })}\left(F\left(\overset{¯}{x}\right)\right)}\left(\nabla F\left(\overset{¯}{x}\right)u^{k}+v^{k}\right)$ where $\overset{¯}{\nu }=\nu^{k,1}$ is the index vector returned from Algorithm 1. Now choosing $\nu^{2},\ldots ,\nu^{K}$ such that $\left(\overset{¯}{\nu },\nu^{2},\ldots ,\nu^{K}\right)\in Q\left(\overset{¯}{x}\right)$ we can simply check by testing $-\nabla f\left(\overset{¯}{x}\right)\in N_{D(\nu^{l})}\left(F\left(\overset{¯}{x}\right)\right)$, l=2,…,K, whether $\overset{¯}{x}$ is $Q_{M}$ stationary or $\overset{¯}{x}$ is not B-stationary.

Further, we have the following corollary.

Corollary 5: Let $\overset{¯}{x}$ be B-stationary for the MPDC (1) and assume that GGCQ is fulfilled at $\overset{¯}{x}$. Let $\overset{¯}{\nu }$ be the index vector returned by Algorithm 1 applied to (36). Then $\overset{¯}{\nu }\in J(F(\overset{¯}{x}))$ and for every $\nu^{2},\ldots ,\nu^{K}$ with $\left(\overset{¯}{\nu },\nu^{2},\ldots ,\nu^{K}\right)\in Q\left(\overset{¯}{x}\right)$ the point $\overset{¯}{x}$ is $Q_{M}$-stationary with respect to $\left(\overset{¯}{\nu },\nu^{2},\ldots ,\nu^{K}\right)$.

## 7. Numerical aspects

In practice, the point $\overset{¯}{x}$ which should be checked for M-stationarity and $Q_{M}$-stationarity, respectively, often is not known exactly. E.g. $\overset{¯}{x}$ can be the limit point of a sequence generated by some numerical method for solving MPDC. Hence, let us assume that we are given some point $\tilde{x}$ close to $\overset{¯}{x}$ and we want to state some rules when we can consider $\tilde{x}$ as approximately M-stationary or $Q_{M}$-stationary. Let us assume that the convex polyhedral sets $D_{i}^{j}$ have the representation

$$\begin{array}{c} D_{i}^{j}=\left\{y\;|\;\left\langle a_{l}^{i,j},y\right\rangle \leq b_{l}^{i,j},\;l=1,\ldots ,p^{i,j}\right\},\;i=1,\ldots ,m_{D},\;j=1,\ldots ,K_{i}, \end{array}$$

where without loss of generality we assume $\|a_{l}^{i,j}\|=1$.

We use here the following approach.


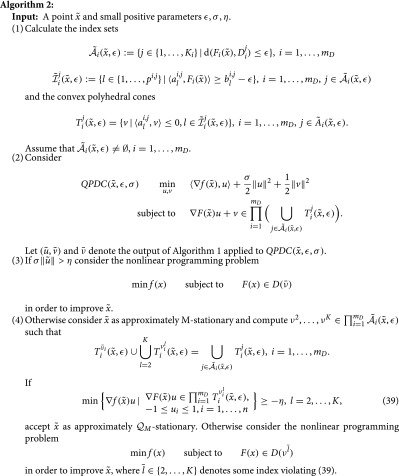


In the first step of Algorithm 2, we want to estimate the tangent cone $T_{D}\left(F\left(\overset{¯}{x}\right)\right)$. In fact, to calculate $T_{D}\left(F\left(\overset{¯}{x}\right)\right)$ we need not to know the point $F(\overset{¯}{x})$, we only need the index sets of constraints active at $\overset{¯}{x}$ and these index sets are approximated by ϵ-active constraints. Note that whenever ${\tilde{A}}_{i}\left(\tilde{x},\epsilon \right)={\tilde{A}}_{i}\left(\overset{¯}{x},0\right)=A_{i}\left(F\left(\overset{¯}{x}\right)\right)$ and ${\tilde{I}}_{i}^{j}\left(\tilde{x},\epsilon \right)={\tilde{I}}_{i}^{j}\left(\overset{¯}{x},0\right)$, $i=1,\ldots ,m_{D}$, $j\in A_{i}\left(F\left(\overset{¯}{x}\right)\right)$ this approach yields the exact tangent cones $T_{D_{i}^{j}}\left(F\left(\overset{¯}{x}\right)\right)=T_{i}^{j}\left(\tilde{x},\epsilon \right)$ for all $i=1,\ldots ,m_{D}$, $j\in A_{i}\left(F\left(\overset{¯}{x}\right)\right)$. To be consistent with the notation of Section 4 we make the convention that in this case the index vector $\overset{¯}{\nu }$ computed in step (2) belongs to $J(\overset{¯}{x})$ and also, whenever we determine $\nu^{2},\ldots \nu^{K}$ is step (4), we have $\left(\overset{¯}{\nu },\nu^{2},\ldots ,\nu^{K}\right)\in Q\left(\overset{¯}{x}\right)$. The regularization term $\frac{\sigma }{2}{\left\|u\right\|}^{2}$ in $QPDC(\tilde{x},\epsilon ,\sigma )$ forces the objective to be strictly convex and therefore Algorithm 1 will always terminate with a Q-stationary solution. Further note that the verification of (39) requires the solution of K-1 linear optimization problems.

The following theorem justifies Algorithm 2. In the sequel, we denote by $M(\overset{¯}{x})$ $\left(M_{sub}\left(\overset{¯}{x}\right)\right)$ the set of all $\nu \in J(\overset{¯}{x})$ such that the mapping F(·)-D(ν) is metrically regular near $\left(\overset{¯}{x},0\right)$ (metrically subregular at $\left(\overset{¯}{x},0\right)$).

Theorem 7: Let $\overset{¯}{x}$ be feasible for the MPDC (1) and assume that ∇f and ∇F are Lipschitz near $\overset{¯}{x}$. Consider sequences $x_{t}\to \overset{¯}{x}$, $\epsilon_{t}↓0$, $\sigma_{t}↓0$ and $\eta_{t}↓0$ with

$$\begin{array}{c} \lim_{t\to \infty }\frac{\|x_{t}-\overset{¯}{x}\|}{\epsilon_{t}}=\lim_{t\to \infty }\frac{\frac{\sigma_{t}}{\eta_{t}}+\left\|x_{t}-\overset{¯}{x}\right\|}{\eta_{t}}=0 \end{array}$$

and let $\left({\tilde{u}}_{t},{\tilde{v}}_{t}\right)$, ${\overset{¯}{\nu }}^{t}$ and eventually $\nu^{t,2}\ldots ,\nu^{t,K_{t}}$ and ${\overset{¯}{l}}_{t}$ denote the output of Algorithm 2 with input data $\left(x_{t},\epsilon_{t},\sigma_{t},\eta_{t}\right)$.

(i) For all *t* sufficiently large and for all $i\in \{1,\ldots ,m_{D}\}$ we have
  (39) $$\begin{array}{c} {\tilde{A}}_{i}\left(x_{t},\epsilon_{t}\right)=A_{i}\left(F\left(\overset{¯}{x}\right)\right),\;{\tilde{I}}_{i}^{j}\left(x_{t},\epsilon_{t}\right)={\tilde{I}}_{i}^{j}\left(\overset{¯}{x},0\right),\;j\in A_{i}\left(F\left(\overset{¯}{x}\right)\right). \end{array}$$
(ii) Assume that the mapping x⇉F(x)-D is metrically regular near $\left(\overset{¯}{x},0\right)$.
  (a) If $\overset{¯}{x}$ is B-stationary then for all *t* sufficiently large the point $x_{t}$ is accepted as approximately M-stationary and approximately $Q_{M}$-stationary.
  (b) If for infinitely many *t* the point $x_{t}$ is accepted as approximately M-stationary then $\overset{¯}{x}$ is M-stationary.
  (c) If for infinitely many *t* the point $x_{t}$ is accepted as approximately $Q_{M}$-stationary and $\left\{{\overset{¯}{\nu }}^{t},\nu^{t,2},\ldots ,\nu^{t,K_{t}}\right\}\subset M\left(\overset{¯}{x}\right)$ then $\overset{¯}{x}$ is $Q_{M}$-stationary.
  (d) For every *t* sufficiently large such that the point $x_{t}$ is not accepted as approximately M-stationary and ${\overset{¯}{\nu }}^{t}\in M_{sub}\left(\overset{¯}{x}\right)$ we have $\min\left\{f\left(x\right)\;|\;F\left(x\right)\in D\left({\overset{¯}{\nu }}^{t}\right)\right\}<f\left(\overset{¯}{x}\right)$.
  (e) For every *t* sufficiently large such that the point $x_{t}$ is not accepted as approximately $Q_{M}$-stationary and $\nu^{t,{\overset{¯}{l}}_{t}}\in M_{sub}\left(\overset{¯}{x}\right)$ we have $\min\left\{f\left(x\right)\;|\;F\left(x\right)\in D\left(\nu^{t,{\overset{¯}{l}}_{t}}\right)\right\}<f\left(\overset{¯}{x}\right)$.

**Proof **(i) Let R>0 be chosen such that *f*, *F* and their derivatives are Lipschitz on $B(\overset{¯}{x},R)$ with constant *L*. It is easy to see that we can choose ϵ>0 such that for all $i\in \{1,\ldots ,m_{D}\}$ we have ${\tilde{A}}_{i}\left(\overset{¯}{x},\epsilon \right)={\tilde{A}}_{i}\left(\overset{¯}{x},0\right)=A_{i}\left(F\left(\overset{¯}{x}\right)\right)$ and such that for every $j\in A_{i}\left(F\left(\overset{¯}{x}\right)\right)$ we have ${\tilde{I}}_{i}^{j}\left(\overset{¯}{x},\epsilon \right)={\tilde{I}}_{i}^{j}\left(\overset{¯}{x},0\right)$. Consider *t* with $\|x_{t}-\overset{¯}{x}\|<R$, $L\|x_{t}-\overset{¯}{x}\|<\epsilon_{t}<\epsilon /2$ and fix $i\in \{1,\ldots ,m_{D}\}$. For every $j\in A_{i}\left(F\left(\overset{¯}{x}\right)\right)$ we have

$$\begin{array}{c} d\left(F_{i}\left(x_{t}\right),D_{i}^{j}\right)\leq \|F_{i}\left(x_{t}\right)-F_{i}\left(\overset{¯}{x}\right)\left\|\leq L\right\|x_{t}-\overset{¯}{x}\|<\epsilon_{t}, \end{array}$$

whereas for $j\notin A_{i}\left(F\left(\overset{¯}{x}\right)\right)$ we have

$$\begin{array}{c} d\left(F_{i}\left(x_{t}\right),D_{i}^{j}\right)\geq d\left(F_{i}\left(\overset{¯}{x}\right),D_{i}^{j}\right)-\|F_{i}\left(x_{t}\right)-F_{i}\left(\overset{¯}{x}\right)\left\|\geq \epsilon -L\right\|x_{t}-\overset{¯}{x}\|>\epsilon_{t} \end{array}$$

showing ${\tilde{A}}_{i}\left(x_{t},\epsilon_{t}\right)=A_{i}\left(F\left(\overset{¯}{x}\right)\right)$. Now fix $j\in A_{i}\left(F\left(\overset{¯}{x}\right)\right)$ and let $l\in {\tilde{I}}_{i}^{j}\left(\overset{¯}{x},0\right)$, i.e. $\left\langle a_{l}^{i,j},F_{i}\left(\overset{¯}{x}\right)\right\rangle =b_{l}^{i,j}$. By taking into account $\|a_{l}^{i,j}\|=1$ we obtain

$$\begin{array}{c} \left\langle a_{l}^{i,j},F_{i}\left(x_{t}\right)\right\rangle \geq b_{l}^{i,j}-\left\|F_{i}\left(x_{t}\right)-F_{i}\left(\overset{¯}{x}\right)\right\|>b_{l}^{i,j}-\epsilon_{t} \end{array}$$

implying $l\in {\tilde{I}}_{i}^{j}\left(x_{t},\epsilon_{t}\right)$, whereas for $l\notin {\tilde{I}}_{i}^{j}\left(\overset{¯}{x},0\right)={\tilde{I}}_{i}^{j}\left(\overset{¯}{x},\epsilon \right)$ we have

$$\begin{array}{c} \left\langle a_{l}^{i,j},F_{i}\left(x_{t}\right)\right\rangle \leq \left\langle a_{l}^{i,j},F_{i}\left(\overset{¯}{x}\right)\right\rangle +\left\|F_{i}\left(x_{t}\right)-F_{i}\left(\overset{¯}{x}\right)\right\|<b_{l}^{i,j}-\epsilon +\epsilon_{t}<b_{l}^{i,j}-\epsilon_{t} \end{array}$$

showing $l\notin {\tilde{I}}_{i}^{j}\left(x_{t},\epsilon_{t}\right)$. Hence, ${\tilde{I}}_{i}^{j}\left(x_{t},\epsilon_{t}\right)=\tilde{I}\left(\overset{¯}{x},0\right)$. Because of our assumptions we have $\|x_{t}-\overset{¯}{x}\|<R$ and $L\|x_{t}-\overset{¯}{x}\|<\epsilon_{t}<\epsilon /2$ for all *t* sufficiently large and this proves (40).

(ii) In view of Proposition 2, we can choose κ large enough such that the mappings F(·)-D, $u⇉\nabla F\left(\overset{¯}{x}\right)u-T_{D}\left(F\left(\overset{¯}{x}\right)\right)$ and F(·)-D(ν), $u⇉\nabla F\left(\overset{¯}{x}\right)u-T_{D(\nu )}\left(F\left(\overset{¯}{x}\right)\right)$, $\nu \in M(\overset{¯}{x})$ are metrically regular near $\left(\overset{¯}{x},0\right)$ with modulus κ. By eventually shrinking *R* we can assume that for every $x\in B(\overset{¯}{x},R)$ the mappings $u⇉\nabla F\left(x\right)u-T_{D}\left(F\left(\overset{¯}{x}\right)\right)$, $u⇉\nabla F\left(x\right)u-T_{D(\nu )}\left(F\left(\overset{¯}{x}\right)\right)$, $\nu \in M(\overset{¯}{x})$ are metrically regular near (0, 0) with modulus κ+1.

Without loss of generality we can assume that $x_{t}\in B\left(\overset{¯}{x},R\right)$ and (40) holds for all *t* implying that $T_{D_{i}^{j}}\left(F\left(\overset{¯}{x}\right)\right)=T_{i}^{j}\left(\tilde{x},\epsilon_{t}\right)$ holds for all $i=1,\ldots ,m_{D}$, $j\in A_{i}\left(F\left(\overset{¯}{x}\right)\right)$. In fact, then the problem $QPDC(x_{t},\epsilon_{t},\sigma_{t})$ is the same as

$$\begin{array}{c} \min_{u,v}\left\langle \nabla f\left(x_{t}\right),u\right\rangle +\frac{\sigma_{t}}{2}{\left\|u\right\|}^{2}+\frac{1}{2}{\left\|v\right\|}^{2}\;\;\text{subject to}\;\nabla F\left(x_{t}\right)u+v\in T_{D}\left(F\left(\overset{¯}{x}\right)\right). \end{array}$$

The point $\left({\tilde{u}}_{t},{\tilde{v}}_{t}\right)$ is Q-stationary for this program and thus also S-stationary by Theorem 4(ii) and the full rank property of the matrix $\left(\nabla F\left(x_{t}\right)\;\vdots \;I\right)$. Hence, there is a multiplier $\lambda_{t}\in {\hat{N}}_{T_{D}\left(F\left(\overset{¯}{x}\right)\right)}\left(\nabla F\left(x_{t}\right){\tilde{u}}_{t}+{\tilde{v}}_{t}\right)\subset N_{T_{D}\left(F\left(\overset{¯}{x}\right)\right)}\left(0\right)$ fulfilling ${\tilde{v}}_{t}+\lambda_{t}=0$, $\nabla f\left(x_{t}\right)+\sigma_{t}{\tilde{u}}_{t}+\nabla F{\left(x_{t}\right)}^{T}\lambda_{t}=0$ and we conclude

(40) $$\begin{array}{c} \|{\tilde{v}}_{t}\left\|=\right\|\lambda_{t}\|\leq \left(\kappa +1\right)\|\nabla f\left(x_{t}\right)+\sigma_{t}{\tilde{u}}_{t}\| \end{array}$$

from (18).

By Q-stationarity of $\left({\tilde{u}}_{t},{\tilde{v}}_{t}\right)$ we know that $\left({\tilde{u}}_{t},{\tilde{v}}_{t}\right)$ is the unique solution of the strictly convex quadratic program

(41) $$\begin{array}{c} \min\left\langle \nabla f\left(x_{t}\right),u\right\rangle +\frac{\sigma_{t}}{2}{\left\|u\right\|}^{2}+\frac{1}{2}{\left\|v\right\|}^{2}\;\;\text{subject to}\;\nabla F\left(x_{t}\right)u+v\in T_{D({\overset{¯}{\nu }}^{t})}\left(F\left(\overset{¯}{x}\right)\right). \end{array}$$

For every α≥0, the point $\alpha ({\tilde{u}}_{t},{\tilde{v}}_{t})$ is feasible for this quadratic program and thus α=1 is solution of

$$\begin{array}{c} \min_{\alpha \geq 0}\;\alpha \left\langle \nabla f\left(x_{t}\right),{\tilde{u}}_{t}\right\rangle +\alpha^{2}\left(\frac{\sigma_{t}}{2}\|{\tilde{u}}_{t}{\|}^{2}+\frac{1}{2}{\left\|{\tilde{v}}_{t}\right\|}^{2}\right) \end{array}$$

implying

$$\begin{array}{c} -\left\langle \nabla f\left(x_{t}\right),{\tilde{u}}_{t}\right\rangle =\sigma_{t}\|{\tilde{u}}_{t}{\|}^{2}+{\left\|{\tilde{v}}_{t}\right\|}^{2}. \end{array}$$

Hence,

(42) $$\begin{array}{c} \sigma_{t}\|{\tilde{u}}_{t}\|\leq -\left\langle \nabla f\left(x_{t}\right),\frac{{\tilde{u}}_{t}}{\|{\tilde{u}}_{t}\|}\right\rangle \leq \left\|\nabla f\left(x_{t}\right)\right\| \end{array}$$

and from (41) we obtain

(43) $$\begin{array}{c} \|{\tilde{v}}_{t}\left\|=\right\|\lambda_{t}\|\leq 2\left(\kappa +1\right)\|\nabla f\left(x_{t}\right)\|. \end{array}$$

(a) Assume on the contrary that $\overset{¯}{x}$ is B-stationary but for infinitely many *t* the point $x_{t}$ is not accepted as approximately M-stationary and hence $\|{\tilde{u}}_{t}\|\geq \eta_{t}/\sigma_{t}$. This implies

$$\begin{array}{cc} d(\nabla F\left(\overset{¯}{x}\right)\frac{{\tilde{u}}_{t}}{\|{\tilde{u}}_{t}\|},T_{D}\left(F\left(\overset{¯}{x}\right)\right)) & \leq d\left(\nabla F\left(x_{t}\right)\frac{{\tilde{u}}_{t}}{\|{\tilde{u}}_{t}\|},T_{D}\left(F\left(\overset{¯}{x}\right)\right)\right)+L\|x_{t}-\overset{¯}{x}\|\leq \frac{\|{\tilde{v}}_{t}\|}{\|{\tilde{u}}_{t}\|}+L\left\|x_{t}-\overset{¯}{x}\right\|\\ & \leq 2\left(\kappa +1\right)\|f\left(x_{t}\right)\|\frac{\sigma_{t}}{\eta_{t}}+L\left\|x_{t}-\overset{¯}{x}\right\| \end{array}$$

and by the metric regularity of $u⇉\nabla F\left(\overset{¯}{x}\right)u-T_{D}\left(F\left(\overset{¯}{x}\right)\right)$ near (0, 0) we can find ${\hat{u}}_{t}\in \nabla F{\left(\overset{¯}{x}\right)}^{-1}T_{D}\left(F\left(\overset{¯}{x}\right)\right)$ with

$$\begin{array}{c} \|{\hat{u}}_{t}-\frac{{\tilde{u}}_{t}}{\|{\tilde{u}}_{t}\|}\|\leq \kappa \left(2\left(\kappa +1\right)\|f\left(x_{t}\right)\|\frac{\sigma_{t}}{\eta_{t}}+L\left\|x_{t}-\overset{¯}{x}\right\|\right). \end{array}$$

Our choice of the parameters $\sigma_{t}$, $\eta_{t}$ together with (43) ensures that for *t* sufficiently large we have

$$\begin{array}{cc} \left\langle \nabla f\left(\overset{¯}{x}\right),{\hat{u}}_{t}\right\rangle & \leq \left\langle \nabla f\left(\overset{¯}{x}\right),\frac{{\tilde{u}}_{t}}{\|{\tilde{u}}_{t}\|}\right\rangle +\left\|\nabla f\left(\overset{¯}{x}\right)\|\right\|{\hat{u}}_{t}-\frac{{\tilde{u}}_{t}}{\|{\tilde{u}}_{t}\|}\|\\ & \leq \left\langle \nabla f\left(x_{t}\right),\frac{{\tilde{u}}_{t}}{\|{\tilde{u}}_{t}\|}\right\rangle +L\|x_{t}-\overset{¯}{x}\|+\|\nabla f\left(\overset{¯}{x}\right)\left\|\right\|{\hat{u}}_{t}-\frac{{\tilde{u}}_{t}}{\|{\tilde{u}}_{t}\|}\|\\ & \leq -\sigma_{t}\|{\tilde{u}}_{t}\left\|+L\right\|x_{t}-\overset{¯}{x}\|+\|\nabla f\left(\overset{¯}{x}\right)\|\|{\hat{u}}_{t}-\frac{{\tilde{u}}_{t}}{\|{\tilde{u}}_{t}\|}\|\\ & \leq -\eta_{t}+L\|x_{t}-\overset{¯}{x}\|+\|\nabla f\left(\overset{¯}{x}\right)\|\kappa \left(2\left(\kappa +1\right)\|f\left(x_{t}\right)\|\frac{\sigma_{t}}{\eta_{t}}+L\left\|x_{t}-\overset{¯}{x}\right\|\right)<0 \end{array}$$

which contradicts B-stationarity of $\overset{¯}{x}$. Hence, for all *t* sufficiently large the point $x_{t}$ must be accepted as approximately M-stationary.

To prove the statement that $x_{t}$ is also accepted as approximately $Q_{M}$-stationary for all *t* sufficiently large we can proceed in a similar way. Assume on the contrary that $\overset{¯}{x}$ is B-stationary but for infinitely many *t* the point $x_{t}$ is not accepted as approximately $Q_{M}$-stationary. For those *t*, let $w_{t}$ denote some element fulfilling $\nabla F\left(x_{t}\right)w_{t}\in T_{D(\nu^{t,{\overset{¯}{l}}_{t}})}\subset T_{D}\left(F\left(\overset{¯}{x}\right)\right)$, $\|w_{t}{\|}_{\infty }\leq 1$ and $\left\langle \nabla f\left(x_{t}\right),w_{t}\right\rangle \leq -\eta_{t}.$ Then, similar as before we can find ${\hat{w}}_{t}\in \nabla F{\left(\overset{¯}{x}\right)}^{-1}T_{D}\left(F\left(\overset{¯}{x}\right)\right)$ such that

$$\begin{array}{c} \|{\hat{w}}_{t}-w_{t}\|\leq \kappa \|\nabla F\left(\overset{¯}{x}\right)-\nabla F\left(x_{t}\right)\left\|\right\|w_{t}\|\leq \kappa L\sqrt{n}\left\|x_{t}-\overset{¯}{x}\right\| \end{array}$$

and for large *t* we obtain

$$\begin{array}{cc} \left\langle \nabla f\left(\overset{¯}{x}\right),{\hat{w}}_{t}\right\rangle & \leq \left\langle \nabla f\left(x_{t}\right),w_{t}\right\rangle +\|\nabla f\left(\overset{¯}{x}\right)-\nabla f\left(x_{t}\right)\left\|\right\|w_{t}\|+\|\nabla f\left(\overset{¯}{x}\right)\left\|\right\|{\hat{w}}_{t}-w_{t}\|\\ & \leq -\eta_{t}+L\sqrt{n}\left(1+\kappa \|\nabla f\left(\overset{¯}{x}\right)\|)\right\|x_{t}-\overset{¯}{x}\|<0 \end{array}$$

contradicting B-stationarity of $\overset{¯}{x}$.

(b) By passing to a subsequence we can assume that for all *t* the point $x_{t}$ is accepted as approximately M-stationary and hence $\sigma_{t}\left\|u_{t}\right\|\leq \eta_{t}\to 0$. By (44) we have that the sequence $\lambda_{t}\in N_{T_{D}\left(F\left(\overset{¯}{x}\right)\right)}\left(0\right)$ is uniformly bounded and by passing to a subsequence once more we can assume that it converges to some $\overset{¯}{\lambda }\in N_{T_{D}\left(F\left(\overset{¯}{x}\right)\right)}\left(0\right)$. By [11, Proposition 6.27] we have $\overset{¯}{\lambda }\in N_{D}\left(F\left(\overset{¯}{x}\right)\right)$ and together with

$$\begin{array}{c} 0=\lim_{t\to \infty }(\nabla f\left(x_{t}\right)+\nabla F{\left(x_{t}\right)}^{T}\lambda_{t})=\nabla f\left(\overset{¯}{x}\right)+\nabla F{\left(\overset{¯}{x}\right)}^{T}\overset{¯}{\lambda } \end{array}$$

M-stationarity of $\overset{¯}{x}$ is established.

(c) By passing to a subsequence we can assume that for all *t* the point $x_{t}$ is accepted as approximately $Q_{M}$-stationary and $\left\{{\overset{¯}{\nu }}^{t},\nu^{t,2},\ldots ,\nu^{t,K_{t}}\right\}\subset M\left(\overset{¯}{x}\right)$. Hence, for all *t* the point $x_{t}$ is also accepted as M-stationary and by passing to a subsequence and arguing as in (b) we can assume that $\lambda_{t}$ converges to some $\overset{¯}{\lambda }\in N_{D}\left(F\left(\overset{¯}{x}\right)\right)$ fulfilling $\nabla f\left(\overset{¯}{x}\right)+\nabla F{\left(\overset{¯}{x}\right)}^{T}\overset{¯}{\lambda }=0$. Since the set $M(\overset{¯}{x})$ is finite, by passing to a subsequence once more we can assume that there is a number *K* and elements $\overset{¯}{\nu },\nu^{2},\ldots ,\nu^{K}$ such that $K_{t}=K$ , ${\overset{¯}{\nu }}^{t}=\overset{¯}{\nu }$ and $\nu^{t,l}=\nu^{l}$, l=2,…,K holds for all *t*. Since we assume that (40) holds we have $\left(\overset{¯}{\nu },\nu^{2},\ldots ,\nu^{K}\right)\in Q\left(\overset{¯}{x}\right)$ and we will now show that $\overset{¯}{x}$ is $Q_{M}$-stationary with respect to $\left(\overset{¯}{\nu },\nu^{2},\ldots ,\nu^{K}\right)$. Since $\left({\tilde{u}}_{t},{\tilde{v}}_{t}\right)$ also solves (42), it follows that $\lambda_{t}=-v_{t}\in N_{T_{D(\overset{¯}{\nu })}\left(F\left(\overset{¯}{x}\right)\right)}\left(\nabla F\left(x_{t}\right){\tilde{u}}_{t}+{\tilde{v}}_{t}\right)\subset N_{D(\overset{¯}{\nu })}\left(F\left(\overset{¯}{x}\right)\right)$ and thus $\overset{¯}{\lambda }\in N_{D}\left(F\left(\overset{¯}{x}\right)\right)\cap N_{D(\overset{¯}{\nu })}\left(F\left(\overset{¯}{x}\right)\right)$ implying $-\nabla f\left(\overset{¯}{x}\right)\in \nabla F{\left(\overset{¯}{x}\right)}^{T}(N_{D}\left(F\left(\overset{¯}{x}\right)\right)\cap (T_{D(\overset{¯}{\nu })}\left(F\left(\overset{¯}{x}\right)\right){)}^{\circ })$. There remains to show $-\nabla f\left(\overset{¯}{x}\right)\in (T_{D(\nu^{l})}\left(F\left(\overset{¯}{x}\right)\right){)}^{\circ }=N_{D(\nu^{l})}\left(F\left(\overset{¯}{x}\right)\right)$, l=2,…,K. Assume on the contrary that $-\nabla f\left(\overset{¯}{x}\right)\notin (T_{D(\nu^{\overset{¯}{l}})}\left(F\left(\overset{¯}{x}\right)\right){)}^{\circ }$ for some index $\overset{¯}{l}\in \{2,\ldots ,K\}$. Then there is some $u\in \nabla F{\left(\overset{¯}{x}\right)}^{-1}T_{D(\nu^{\overset{¯}{l}})}\left(F\left(\overset{¯}{x}\right)\right)$, ${\left\|u\right\|}_{\infty }=\frac{1}{2}$ such that $\langle \nabla f(\overset{¯}{x}),u\rangle =:-\gamma <0$ and since $\nu^{\overset{¯}{l}}\in M\left(\overset{¯}{x}\right)$, for each *t* there is some ${\hat{u}}_{t}\in \nabla F{\left(x_{t}\right)}^{-1}T_{D(\nu^{\overset{¯}{l}})}\left(F\left(\overset{¯}{x}\right)\right)$ with

$$\|u-{\hat{u}}_{t}\|\leq \left(\kappa +1\right)\|\nabla F\left(\overset{¯}{x}\right)-\nabla F\left(x_{t}\right)\left\|\|u\right\|\leq \frac{\sqrt{n}}{2}\left(\kappa +1\right)L\left\|x_{t}-\overset{¯}{x}\right\|.$$

It follows that for all *t* sufficiently large we have $\|{\hat{u}}_{t}{\|}_{\infty }\leq 1$ and

$$\begin{array}{cc} \left\langle \nabla f\left(x_{t}\right),{\hat{u}}_{t}\right\rangle & \leq \left\langle \nabla f\left(\overset{¯}{x}\right),u\right\rangle +\|\nabla f\left(x_{t}\right)-\nabla f\left(\overset{¯}{x}\right)\left\|\right\|{\hat{u}}_{t}\|+\|\nabla f\left(\overset{¯}{x}\right)\|\|u-{\hat{u}}_{t}\|\\ & \leq -\gamma +L\sqrt{n}\left(1+\frac{\kappa +1}{2}\right)\left\|x_{t}-\overset{¯}{x}\right\|<-\eta_{t} \end{array}$$

contradicting our assumption that $x_{t}$ is accepted as approximately $Q_{M}$-stationary.

(d), (e) We assume that κ is chosen large enough such that the mappings F(·)-D(ν), $\nu \in M_{sub}\left(\overset{¯}{x}\right)$ are metrically subregular at $\left(\overset{¯}{x},0\right)$ with modulus κ. Then by [21, Proposition 2.1] the mappings $u⇉\nabla F\left(\overset{¯}{x}\right)u-T_{D(\nu )}\left(F\left(\overset{¯}{x}\right)\right)$, $\nu \in M_{sub}\left(\overset{¯}{x}\right)$ are metrically subregular at (0, 0) with modulus κ as well. Taking into account that $\left({\tilde{u}}_{t},{\tilde{v}}_{t}\right)$ solves (42), we can copy the arguments from part (a) with $T_{D}\left(F\left(\overset{¯}{x}\right)\right)$ replaced by $T_{D({\overset{¯}{\nu }}^{t})}\left(F\left(\overset{¯}{x}\right)\right)$ to show the existence of ${\hat{u}}_{t}\in \nabla F{\left(\overset{¯}{x}\right)}^{-1}T_{D({\overset{¯}{\nu }}^{t})}\left(F\left(\overset{¯}{x}\right)\right)$ with $\langle \nabla f\left(\overset{¯}{x}\right),{\hat{u}}_{t}\rangle <0$ whenever $x_{t}$ is not accepted as approximately M-stationary and *t* is sufficiently large. In doing so, we also have to recognize that metric regularity of $u⇉\nabla F\left(\overset{¯}{x}\right)u-T_{D({\overset{¯}{\nu }}^{t})}\left(F\left(\overset{¯}{x}\right)\right)$ can be replaced by the weaker property of metric subregularity. Since ${\overset{¯}{\nu }}^{t}\in M_{sub}\left(\overset{¯}{x}\right)$, ${\hat{u}}_{t}$ is a feasible descent direction and for sufficiently small α>0 the projection of $\overset{¯}{x}+\alpha {\hat{u}}_{t}$ on $F^{-1}\left(D\left({\overset{¯}{\nu }}^{t}\right)\right)$ yields a point with a smaller objective function value than $\overset{¯}{x}$. This proves (d). In order to show (e), we can proceed in a similar way. Using the same arguments as in part (a), we can prove the existence of a feasible direction ${\hat{w}}_{t}\in T_{D(\nu^{t,{\overset{¯}{l}}_{t}})}$ with $\langle \nabla f\left(\overset{¯}{x}\right),{\hat{w}}_{t}\rangle <0$, whenever *t* is sufficiently large and $x_{t}$ is not accepted as approximately $Q_{M}$-stationary. Together with $\nu^{t,{\overset{¯}{l}}_{t}}\in M_{sub}\left(\overset{¯}{x}\right)$ the assertion follows.
